# Landscape of cohesin-mediated chromatin loops in the human genome

**DOI:** 10.1038/s41586-020-2151-x

**Published:** 2020-07-29

**Authors:** Fabian Grubert, Rohith Srivas, Damek V Spacek, Maya Kasowski, Mariana Ruiz-Velasco, Nasa Sinnott-Armstrong, Peyton Greenside, Anil Narasimha, Qing Liu, Benjamin Geller, Akshay Sanghi, Michael Kulik, Silin Sa, Marlene Rabinovitch, Anshul Kundaje, Stephen Dalton, Judith B. Zaugg, Michael Snyder

**Affiliations:** 1 Department of Genetics, Stanford University School of Medicine, Palo Alto, CA USA; 2 Department of Pathology, Stanford University School of Medicine, Palo Alto, CA USA; 3 Structural and Computational Biology, European Molecular Biology Laboratory, Heidelberg, Germany; 4 Biomedical Informatics Graduate Training Program, Stanford University School of Medicine, Stanford, CA USA; 5 Center for Molecular Medicine, University of Georgia, Athens, GA USA; 6 Department of Biochemistry and Molecular Biology, University of Georgia, Athens, GA USA; 7 Vera Moulton Wall Center for Pulmonary Vascular Diseases, Stanford University School of Medicine, Palo Alto, CA USA; 8 Stanford Cardiovascular Institute, Stanford University School of Medicine, Palo Alto, CA USA; 9 Department of Pediatrics, Stanford University School of Medicine, Palo Alto, CA USA; 10 Department of Computer Science, Stanford University, Stanford, CA USA

**Keywords:** Chromatin immunoprecipitation, Chromatin structure

## Abstract

Physical interactions between distal regulatory elements have a key role in regulating gene expression, but the extent to which these interactions vary between cell types and contribute to cell-type-specific gene expression remains unclear. Here, to address these questions as part of phase III of the Encyclopedia of DNA Elements (ENCODE), we mapped cohesin-mediated chromatin loops, using chromatin interaction analysis by paired-end tag sequencing (ChIA-PET), and analysed gene expression in 24 diverse human cell types, including core ENCODE cell lines. Twenty-eight per cent of all chromatin loops vary across cell types; these variations modestly correlate with changes in gene expression and are effective at grouping cell types according to their tissue of origin. The connectivity of genes corresponds to different functional classes, with housekeeping genes having few contacts, and dosage-sensitive genes being more connected to enhancer elements. This atlas of chromatin loops complements the diverse maps of regulatory architecture that comprise the ENCODE Encyclopedia, and will help to support emerging analyses of genome structure and function.

## Abstract (web summary)

A map of cohesin-mediated chromatin loops in 24 types of human cells identifies loops that show cell-type-specific variation, indicating that chromatin loops may help to specify cell-specific gene expression programs and functions.

## Main

The way in which the genome is organized at different scales is a longstanding topic of investigation. The development of high-throughput chromatin conformation assays (for example, Hi-C^1^ and ChIA-PET^2^) has substantially furthered our understanding of the 3D organization of the human genome and how it influences gene regulation. Topologically associating domains (TADs) have been identified as a fundamental structural and regulatory unit of the genome^3–5^. These megabase-scale contiguous regions are characterized by a high density of self-interactions, often between distal enhancers and promoters. These domains both promote long-range gene regulatory interactions within their boundaries and insulate enhancers from neighbouring domains to prevent ectopic activity^3–11^. The locations of TADs are specified in part by CTCF binding sites, which arrest loop extrusion through the ring-like cohesin complex^12–16^. Disruption of TAD boundaries and chromatin loops has been associated with human diseases, including congenital limb malformations and cancer, through a mechanism involving alterations in enhancer–gene interactions^8,11,17^.

Previous work has established that the locations of TAD boundaries are largely invariant across cell types and species and during cell differentiation, consistent with the idea that these domains have a constrained role in organizing the genome^3,18,19^. Recent findings, however, have suggested that TADs are further organized into sub-TADs, which vary in both strength of interaction and location, and may be important for the determination and maintenance of cell fates^12,18,20,21^. However, the extent to which these sub-TADs and the cohesin-mediated chromatin loops that define them vary and influence differences in gene expression among cell types has not been fully characterized. More generally, the role of cohesin-mediated loops in gene regulation is not well understood. Our goal was to characterize the extent of variation in cohesin-mediated chromatin loops across human cell types.

We have used the ChIA-PET assay to map cohesin-bound chromatin loops and quantify their frequency across 24 cell types^2,22,23^ (including core ENCODE cell lines) that span all three germ layers, including multiple embryonic cell lines and primary cell types (Supplementary Table 1). About 28% of all loops vary among the investigated cell types, and these differences are effective in grouping cell types according to their tissue group of origin (blood, solid tissue or embryonic). We have further integrated our data with RNA expression data and maps of active enhancers (H3K27ac; acetylation at lysine 27 of histone H3) to test whether changes in loops correlate with gene expression differences or splicing, and examine which chromatin states coincide with cell-type-specific loops. Our data serve as a resource for investigating the effect of 3D chromatin interactions on the regulation of gene expression programs that define cell-type identity and for linking disease-relevant regulatory elements to potential target genes. Specific highlights of our findings are given below.

- We used the ChIA-PET assay to map cohesin-bound chromatin loops and quantify their interaction frequency across 24 cell types.
- Analyses of loop interaction frequencies in our data set effectively grouped cell types, including those derived from the same individual, according to their tissue group of origin. The groupings are concordant across gene regulatory phenotypes, suggesting that loop variation recapitulates cell-type identity in a similar manner to enhancer activity and gene expression, and is mainly driven by epigenetic factors.
- We found that approximately one-quarter of cohesin-mediated chromatin loops varied across cell types, showing substantial variability in interactions at the sub-TAD scale. Variable loops tend to span shorter distances and are depleted in housekeeping genes.
- Approximately one-quarter of cohesin-mediated loops are anchored by enhancers across diverse cell types, representing the most enriched loop-associated chromatin state. Enhancer anchors participate in more interactions than promoter anchors and are enriched for interactions with other enhancers and transcription start sites (TSSs), consistent with groups of enhancer-associated loops regulating promoters.
- Cell-type-specific loops coincide with different chromatin states. For example, stem cell loops show reduced active promoter and transcribed states, and increased bivalent states, which may point to a role of these loops in maintaining pluripotency.
- In our interaction map, genes that have more interactions are depleted for housekeeping functions and enriched for pathogenic variants and haploinsufficiency, suggesting that the connectivity of a gene is linked to its function and role in disease.
- Loop variation modestly correlates with gene expression variation, especially for loops that link an enhancer directly to a promoter; a weaker positive correlation is observed for genes internal to loops and for neighbouring genes within the same loop.
- Group-specific (blood and embryonic) loops show enrichment of cell-type-specific transcription factor (TF) motifs at loop ends and are enriched in genes with group-specific functions. Genome-wide association study (GWAS) variants for autoimmunity are enriched in blood-specific loops, but not in embryonic loops, pointing to the importance of cohesin-mediated loops for understanding the mechanisms of human disease variants.

## Genome-wide map of chromatin interactions

**Extended Data Fig. 1:**
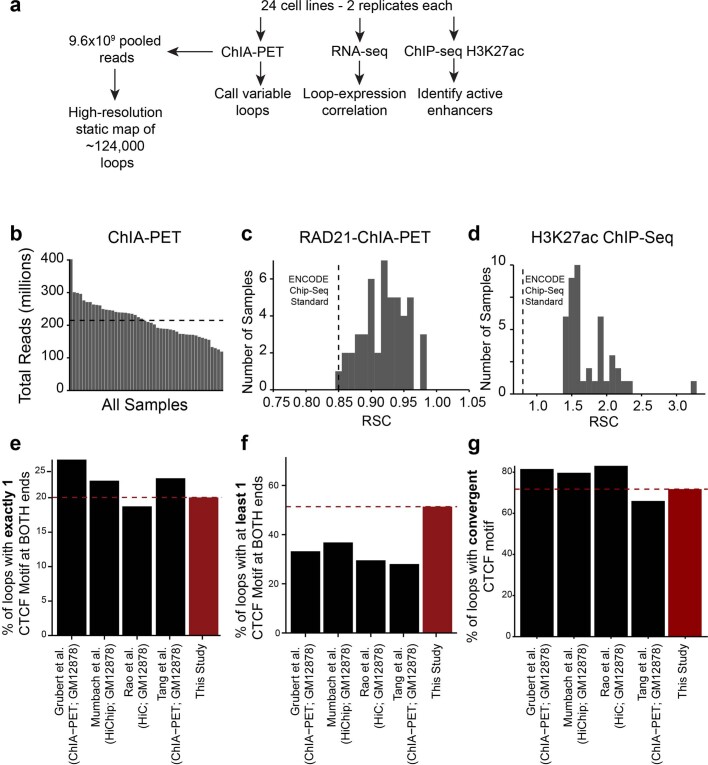
Quality metrics and convergence rates. **a**, Flowchart of study design. **b**, Total number of reads obtained for each ChIA-PET sample **c**, Relative strand correlation (RSC) score for RAD21 ChIA-PET data. **d**, RSC for H3K27ac ChIP–seq data. **e**–**g**, Comparison of CTCF motif presence and orientation at chromatin loops identified in this study and other published data sets. **e**, Fraction of chromatin loops with exactly one CTCF motif at both loop ends. **f**, Fraction of chromatin loops with at least one CTCF motif at both loop ends. **g**, Fraction of chromatin loops with convergent CTCF motif orientation.

To identify cell-type-specific chromatin loops on a genome-wide scale, we generated 3D chromatin interaction maps at single-cohesin peak resolution (about 2-kb) using a modified ChIA-PET assay (Extended Data Fig. 1a, Methods). In brief, this chromatin conformation capture assay incorporates an immunoprecipitation step followed by proximity ligation to measure the frequency of interactions between pairs of genomic regions bound by a protein of interest. We chose the RAD21 subunit of the cohesin complex, which facilitates physical contacts between genes and enhancers^22,24^ and is essential for chromatin loop assembly and subsequent TAD formation^12–14,16^. Henceforth we refer to these cohesin-mediated chromatin loops as loops or interactions^12,23^. We generated a median of about 200 million paired-end reads (2 × 101 bp) per experiment (Extended Data Fig. 1b, Supplementary Table 2). To study the interplay between loops, regulatory elements, and gene expression, we also generated chromatin immunoprecipitation with sequencing (ChIP–seq) data for the histone mark H3K27ac, which demarcates active promoters and enhancers^25,26^, and paired-end RNA sequencing (RNA-seq) data (Supplementary Table 2). The ChIP efficiency for RAD21 and H3K27ac passed ENCODE ChIP–seq quality standards^27^ (Extended Data Fig. 1c, d). All experiments were performed in biological replicates.

**Fig. 1:**
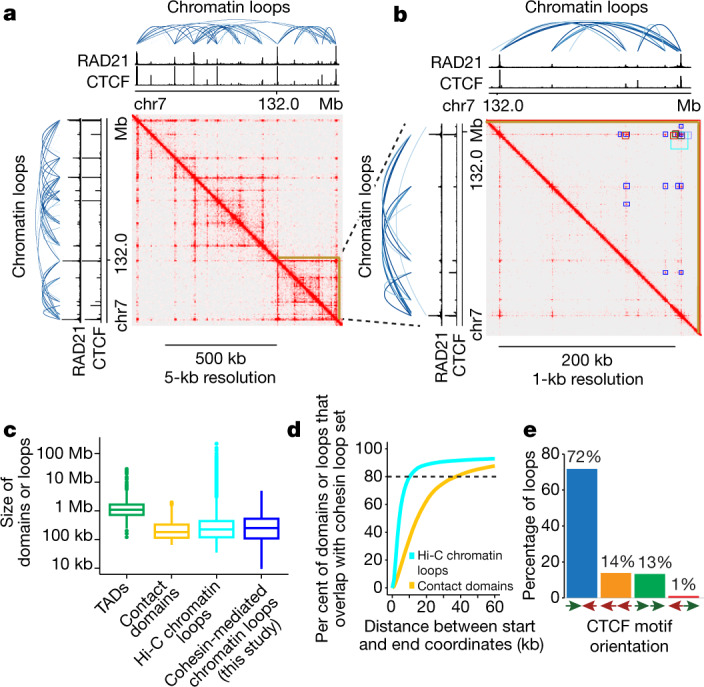
Characteristics of cohesin-mediated chromatin interactions. **a**, **b**, Cohesin ChIA-PET heat maps for the pan-cell-line data set. Signal tracks at the top and to the left of heat maps correspond to CTCF and RAD21 (cohesin) ChIP–seq signals and cohesin ChIA-PET loops (blue). **a**, Approximately 750-kb view including a contact domain (brown triangle) identified in lung fibroblasts (IMR90)^12^. **b**, Approximately 250-kb expanded view of contact domain (brown triangle). Dark blue squares, chromatin loops identified in our data set. For comparison, loops identified with in-situ Hi-C across eight cell lines^12^ are shown as squares in various colours. Heat maps were generated with Juicer^64^ and visualized with Juicebox^65^. **c**, Sizes of cohesin-mediated chromatin loops identified in this study (*n* = 124,830) relative to TADs^19^ (*n* = 35,435), contact domains^12^ (*n* = 9,263), and high-resolution in situ Hi-C chromatin loops^12^ (*n* = 19,846). Centre line represents the median, box extent ranges from 25th to 75th percentile and whiskers extend at most to 1.5× the interquartile range. Summary statistics for the boxplots can be found in Supplementary Table 9. **d**, Per cent of Hi-C chromatin loops across seven cell lines^12^ (light blue) or contact domains from GM12878^12^ (yellow) that overlap our pan-cell-line loop set. **e**, CTCF motif orientation at chromatin loop ends.

To define a comprehensive, high-resolution set of chromatin loops, we pooled ChIA-PET data sets across all 24 cell types in our study (representing about 10 billion reads) and called a unified set of interactions using the Mango pipeline^28^ (Methods), which accounts for various biases, including genomic distance between interacting loci and local ChIP efficiency. This pooled set yielded 124,830 loops (Fig. 1a, b, Supplementary Tables 3, 4, Methods), which represents, to our knowledge, the most comprehensive high-resolution set generated across cell types. These loops are similar in size to the chromatin loops and contact domains that were recently identified by high-resolution in-situ Hi-C^12^ and are about 4–5 times smaller than previously identified TADs^3,19^ (Fig. 1c). Overall, our unified set of loops overlaps with more than 90% of previously identified Hi-C chromatin loops across seven cell lines^12^, and 60% of contact domains for GM12878 cells^12^, respectively (Fig. 1d).

Most loops form between CTCF binding sites oriented in a convergent manner^12^. Consistent with this model, 72% of our loops with CTCF motifs at both ends exhibited convergent motif orientation (Fig. 1e, Methods). This result is robust to varying thresholds used to call loops and is in accordance with previously published sets of chromatin loops identified using different 3C-based assays, such as in-situ Hi-C, ChIA-PET and Hi-ChIP^9,12,23,29^ (Extended Data Fig. 1e–g, Methods).

## Loop variability and cell type

**Fig. 2:**
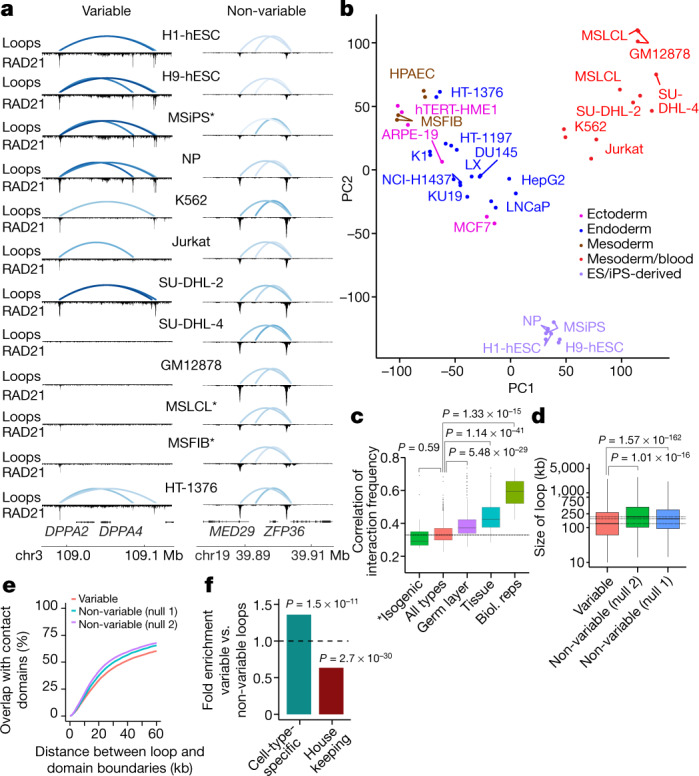
Chromatin loop variation across 24 cell types. **a**, Examples of variable (left) and non-variable loops (right) across cell types. Chromatin loops are displayed above the corresponding RAD21 signal tracks. The colour density of loops corresponds to normalized interaction frequency (darker blue indicates higher frequency). *Isogenic cell types. **b**, PCA of normalized chromatin loop interaction frequencies (*n* = 85,294 loops versus *n* = 48 samples (24 cell types × 2 replicates each)). Colours denote the germ layer origin of each sample (Supplementary Table  2). **c**, Correlation of interaction frequencies between pairs of cell types (all types, *n* = 1,104 pairs; isogenic, *n* = 15; germ layer, *n* = 316; tissue, *n* = 160; biological replicates, *n* = 24; *P* values calculated using two-sided Wilcoxon rank-sum test). Centre line represents the median, box extent ranges from 25th to 75th percentile, and whiskers extend at most to 1.5× the interquartile range. Summary statistics for the boxplots can be found in Supplementary Table 9. **d**, Size distribution of variable chromatin loops versus two different sets of non-variable control loops (*n* = 35,698, significance assessed using two-sided *t*-test). Centre line represents the median, box extent ranges from 25th to 75th percentile, and whiskers extend at most to 1.5× the interquartile range. Summary statistics for the boxplots can be found in Supplementary Table 9. **e**, Overlap of variable and non-variable chromatin loops with contact domains^12^. **f**, Enrichment of cell-type-specific genes and depletion of housekeeping genes (*n* = 2,220) in variable versus non-variable loops (*n* = 35,698). *P* values calculated using a two-sided Fisher’s exact test. Summary statistics for the figure can be found in Supplementary Table 9.

Inspection of our data set revealed two broad classes of loops—those in which the normalized interaction frequencies (the number of paired-end tags (PETs) that link the two ends of a loop) varied across cell types and those that were relatively non-variable (Fig. 2a). For example, both *DPPA2* and *DPPA4* were entirely contained within two loops in the stem cell lines that we used (H1-hESC, H9-hESC, and MSiPS); however, these loops were either absent (for example, in GM12878 and MSLCL cells) or displayed reduced interaction frequency in a number of cancer cell lines (for example, Jurkat and K562 cells). Consistent with this observation, both *DPPA2* and *DPPA4* are active during development^30^ and have been implicated in cancer^31^.

**Extended Data Fig. 2:**
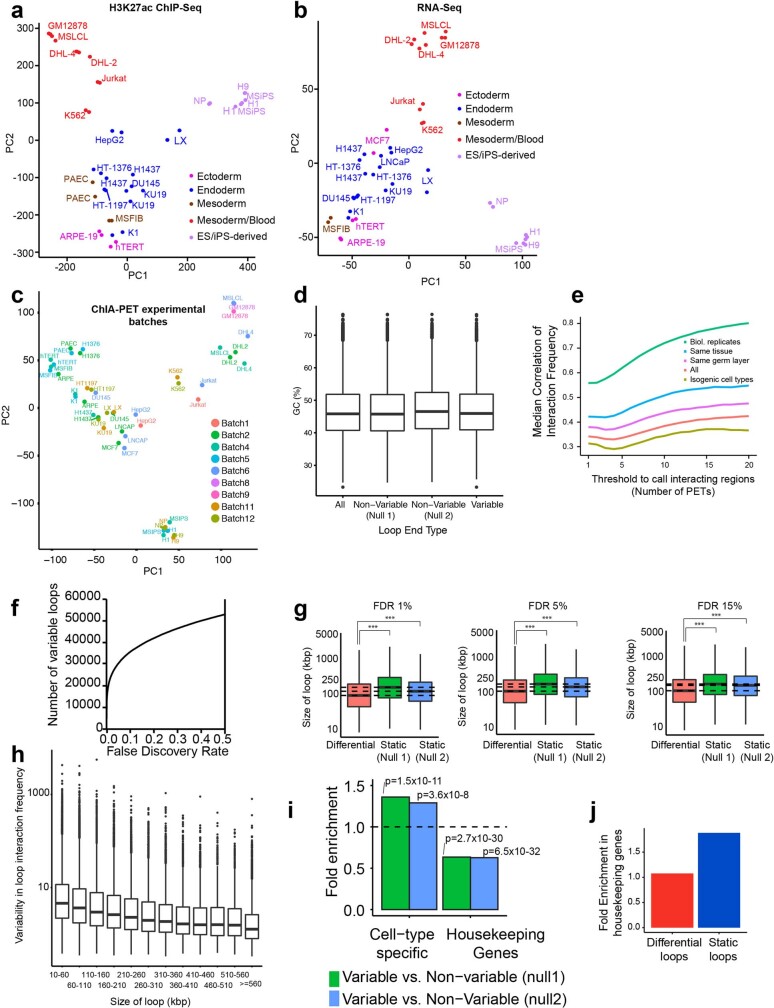
Variability in chromatin loops. **a**, **b**, PCA of H3K27ac ChIP–seq data (**a**; *n* = 288,711 peaks versus *n* = 44 samples (22 cell types × 2 replicates each)) and RNA-seq data (**b**; *n* = 22,197 genes versus *n* = 46 samples (23 cell types × 2 replicates each)); samples are coloured according to the germ layer from which they originated. **c**, PCA of chromatin loop interaction frequencies (*n* = 85,294 loops versus *n* = 48 samples (24 cell types × 2 replicates each)). Colours denote the experimental batch of each sample. **d**, GC content in anchor regions of different sets of chromatin loops. Centre line represents the median, box extent ranges from 25th to 75th percentile and whiskers extend at most to 1.5× the interquartile range. Summary statistics for the boxplots can be found in Supplementary Table 9. **e**, Correlation of chromatin loop interaction frequencies (Spearman rank correlation; *y*-axis) between pairs of cell types at varying PET thresholds (*x*-axis). **f**, Number of variable loops found at different FDR thresholds. **g**, Size distribution of variable chromatin loops versus non-variable loops at different FDR cutoffs. ***P < 0.04. Significance was assessed using a two-sided *t*-test. Centre line represents the median, box extent ranges from 25th to 75th percentile, whiskers extend at most to 1.5× the interquartile range. Summary statistics for the boxplots can be found in Supplementary Table 9. **h**, Variability of loops of different sizes. Summary statistics for all box plots can be found in Supplementary Table 9. **i**, Enrichment of cell-type-specific genes and depletion of housekeeping genes (*n* = 2,220) in variable versus non-variable loops (*n* = 35,698) for null sets 1 and 2. *P* values by Fisher’s exact test. **j**, Enrichment of broadly expressed genes at variable and non-variable chromatin loops. The set of broadly expressed genes was obtained from the GTEx project^33,34^.

We sought to use our measurement of interaction frequencies to systematically identify variable loops across different cell types. First, we subjected normalized interaction frequencies across all cell types to principal component analysis (PCA) (Fig. 2b). All cell types fell into one of three main clusters—blood, stem-cell like (embryonic), and solid-tissue-derived—with 7.3% of variability explained by PC1 and 6.7% by PC2. PCA for the RNA-seq and H3K27ac ChIP–seq data yielded similar clustering patterns (Extended Data Fig. 2a, b). The clusters did not correspond to the batches in which the samples were processed (Extended Data Fig. 2c) and were robust to various data processing choices (Methods). We also checked that the variability was not due to varying GC content in the anchor regions involved (Extended Data Fig. 2d), as well as other technical confounders (Methods). As expected, biological replicates clustered much more closely than different cell types (Fig. 2b, c, Extended Data Fig. 2e, Methods). Two lymphoblastoid cell lines clustered together in the PCA despite the fact that one (GM12878) has been propagated over decades, whereas the other (MSLCL) was recently established^32^, indicating that cohesin looping is conserved during long-term cell passage. Notably, cells from three cell lines (dermal fibroblast (MSFIB), lymphoblastoid (MSLCL), and iPSC (MSiPS)) that were derived from the same donor were each found in one of the three main clusters and displayed the lowest correlated interaction frequencies among those tested (Fig. 2b, c). These results indicate that loop variation among cell types is likely to be driven by epigenetic factors rather than genetic variants and to exceed variation driven by genetic differences among people, particularly for more distantly related cell types.

Having established that normalized interaction frequencies could reliably group related cell types, we next sought to quantify loop variability. We used a linear mixed effects model to identify loops that varied in interaction frequency across our set of 24 cell types (Methods). To test for variability, we filtered loops to include only those with four or more PETs in at least one sample, which yielded 85,294 loops (Supplementary Table 4). At FDR < 10%, we identified 35,698 variable loops, or 41% of all tested loops (28% of the pan-cell line loop set) (Extended Data Fig. 2f, Methods). Variable loops spanned significantly shorter distances than non-variable loops (130 kb versus 178 kb) (Fig. 2d, Extended Data Fig. 2g, h). Variable loops also tended to overlap contact domain boundaries to a lesser degree than non-variable loops (Fig. 2e, Methods).

Finally, we examined whether loop variability is associated with specific types of genes. Using our RNA-seq data, we defined genes as broadly expressed or cell-type-specific (Methods). Overall, variable loops showed enrichment for cell-type-specific gene expression relative to non-variable loops, whereas they were depleted in genes that are expressed across all cell types (Fig. 2f, Extended Data Fig. 2i). In agreement with this observation, non-variable loops were enriched in genes that are broadly expressed across a larger set of tissue types^33,34^ (Extended Data Fig. 2j).

## Cell-type-specific loops and chromatin states

**Fig. 3:**
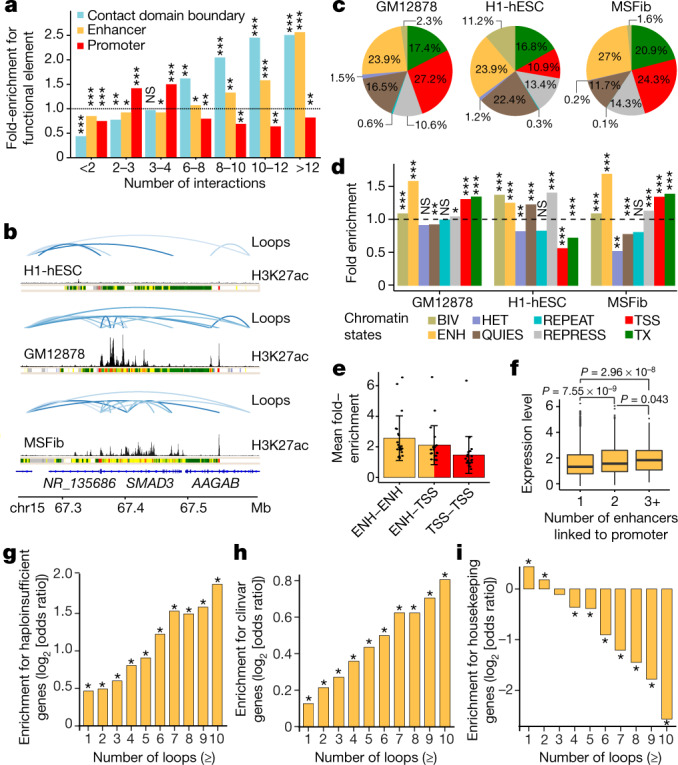
Cell-type-specific loops exhibit enrichment for specific chromatin states. **a**, Enrichment of domain boundaries, enhancers and promoters relative to connectivity of loop ends (number of interactions assessed, 124,830; *P < 0.05, **P < 0.005, ***P < 2.2 × 10^−16^; NS, not significant (*P* = 0.67); significance assessed by two-sided Fisher’s exact test). Summary statistics for the enrichment calculations can be found in Supplementary Table 9. **b**, Examples of cell-type-specific active enhancers and chromatin loops across three cell types. Chromatin loops are displayed above the corresponding H3K27ac signal tracks. Loop colour intensity corresponds to interaction frequency. Below the H3K27ac track is the chromatin state annotation obtained from the Roadmap Epigenomics Mapping Consortium^39^. H1-hESC cells have minimal enhancer activity and few loops. Cohesin loops colocalize with regions of high enhancer activity in GM12878 and MSFib cells. **c**, Proportion of chromatin states in cell-type-specific loop ends for a lymphoblastoid cell line (GM12878), an embryonic line (H1-hESC) and a skin-derived fibroblast line (MSFib). **d**, Fold-enrichments of chromatin states at cell-type-specific loop ends in GM12878, H1-hESC and MSFib cells. Number of interactions assessed (top 10%) = 8,529; *P < 0.05, **P < 0.005, ***P < 2.2 × 10^−16^; NS, not significant; *P* values assessed by two-sided Fisher’s exact test and adjusted for multiple hypothesis testing using the Benjamini–Hochberg procedure. See Supplementary Table 9 for a complete list of enrichments and *P* values. **e**, Fold-enrichments of cell-type-specific loops linking cell-type-specific enhancer–enhancer pairs (ENH–ENH; mean = 2.57), enhancer–promoter pairs (ENH–TSS; mean = 2.18) and promoter–promoter pairs (TSS–TSS; mean = 1.47) (*n* = 21 cell types; error bars, s.d.). **f**, Normalized expression level for each gene, binned by the number of cell-type-specific enhancer connections per gene (*P* values assessed by two-sided Wilcoxon sum-rank test; centre line represents the median, box extent ranges from 25th to 75th percentile and whiskers extend at most to 1.5× the interquartile range. Summary statistics for the boxplots can be found in Supplementary Table 9. **g**–**j**, log_2_[odds ratios] for haploinsufficient genes (**g**), disease genes in ClinVar (**h**), and housekeeping genes (**i**) that have a certain number of enhancers linked to their promoters. *P < 0.05, two-sided Fisher’s exact test with Benjamini–Hochberg adjustment for multiple hypothesis testing; *n* = 19,353 chromatin loops. See Supplementary Table 9 for a complete list of *P* values.

Enhancers often exert their influence on gene expression over large distances through direct 3D chromatin contacts with multiple distal promoters^35–37^. To study the subset of cohesin loops that mediate enhancer contacts, we profiled the enhancer mark H3K27ac using ChIP–seq and quantified signal at 288,711 genomic regions that were enriched for enhancer activity in at least two cell types (‘enhancers’) (Supplementary Table 5, Methods). Loop ends from our pan-cell-type data set showed increasing overlap with enhancer regions the more interactions they were involved in; the same was observed for contact domain boundaries (Fig. 3a). On the other hand, loop ends with few interactions tended to coincide with promoters (this result was robust to the threshold used to define the pan-cell-line loop set (Methods)). Together, these data are consistent with a ‘hub and spoke’ model in which groups of enhancers work together through cohesin-mediated looping to target and regulate multiple promoters^38^.

**Extended Data Fig. 3:**
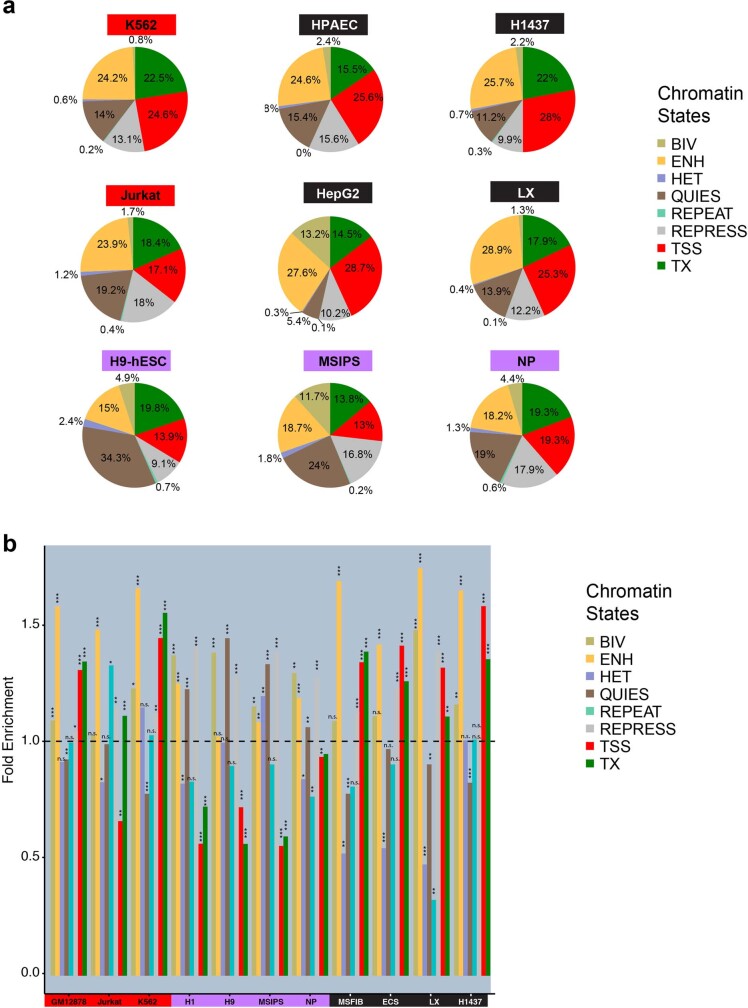
Cell-type-specific loops exhibit enrichment for specific chromatin states. **a**, Proportion of chromatin states in cell-type-specific loop ends for various cell types from the blood group (red), the stem cell/embryonic group (purple) and the group derived from solid tissue (black). **b**, Fold-enrichment of chromatin states in cell-type-specific loop ends for the cell types in **a**. Number of interactions assessed (top 10%) = 8,529; *P < 0.05, **P < 0.005, ***P < 2.2 × 10^−16^, n.s. = non-significant; *P* values assessed by two-sided Fisher’s exact test and corrected for multiple hypothesis testing using the Benjamini–Hochberg procedure. See Supplementary Table 9 for a complete list of enrichments and *P* values.

Manual inspection of our data indicated that cell-type-specific loops tended to overlap with cell-type-specific regulatory elements, such as enhancers (Fig. 3b). Thus, we investigated which chromatin states overlapped cell-type-specific loop ends. To this end, we obtained chromatin state calls from the Roadmap Epigenomics Mapping Consortium^39^ for 12 cell types (Supplementary Table 1). Next, for each cell type, we identified a set of cell-type-specific interactions—loops with high interaction frequencies in the cell of interest, but reduced frequencies in all other cell types (Methods). Finally, we tabulated the number of chromatin state elements across eight categories that overlapped each set of cell-type-specific loop ends (Methods). As in the pan-cell-type data set, in nearly all cell types, genomic elements in the enhancer state (ENH) represented a large proportion (about 23%; Fig. 3c, Extended Data Fig. 3a) of elements that overlapped loop ends. However, in embryonic cell lines, the elements that overlapped loop ends showed a modest increase in inactive bivalent (BIV) and quiescent (QUIES) states, and reduced representation of active TSS-proximal promoter (TSS) and actively transcribed (TX) states. Enrichment tests revealed a reduction in elements in the TSS and TX states in stem cell lines, and a modest increase in elements in the BIV state (Fig. 3d, Extended Data Fig. 3b, Supplementary Table 9), which might be explained by the involvement of chromatin loops in maintaining pluripotency in stem cells by linking bivalent elements^40,41^. These results were robust to the threshold used to define the set of cell-type-specific interactions (Methods).

Next, we investigated whether cell-type-specific cohesin-mediated chromatin loops might specifically connect cell-type-specific enhancers and expressed genes. Similar to past studies that have approached this question using a promoter-centric view^42–44^, we observed strong enrichment for enhancer–promoter (ENH–TSS) interactions (Fig. 3e). In addition, interactions between enhancers (ENH–ENH), but not between promoters (TSS–TSS), were enriched. Studies in different systems have shown that the number of enhancers linked to a given promoter is associated with the RNA expression level^12,42,44^. We looked for this effect in promoters that are linked to enhancers by cohesin-mediated chromatin loops, by binning genes on the basis of the number of linked enhancers; the number of enhancers was modestly but significantly correlated with expression level (Fig. 3f), suggesting that the recruitment of additional physically linked enhancers may help to regulate gene expression. Again, these results were robust to the choice of threshold used to define cell-type-specific interactions (Methods).

## Gene connectivity corresponds to function

**Extended Data Fig. 4:**
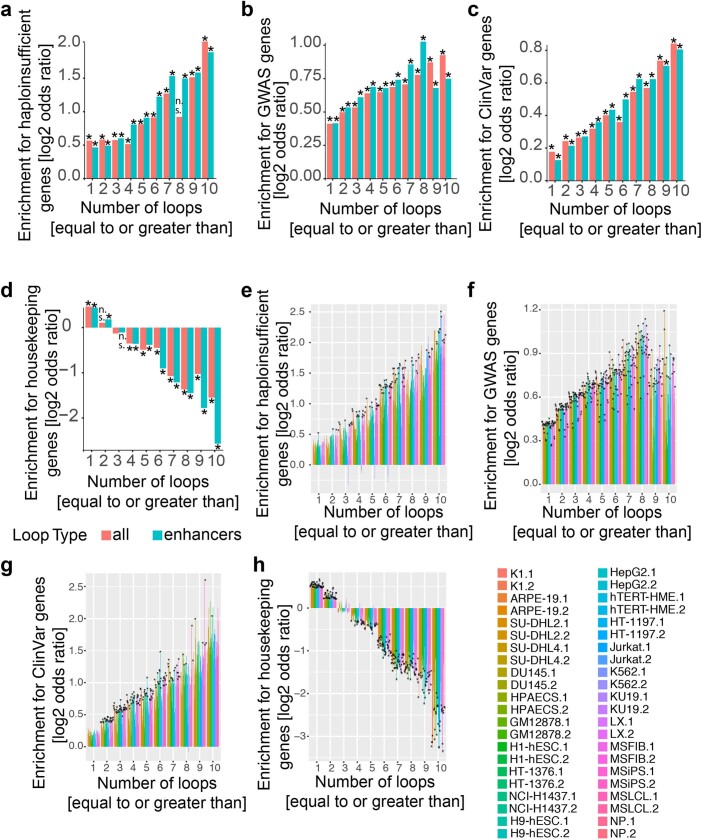
Connectivity of genes corresponds to gene function. **a**–**d**, log_2_ odds ratios for different groups of genes with a certain number of loops linked to their promoters (*adjusted *P* < 0.05 by two-sided Fisher’s exact test; *n* = 19,353 loops). See Supplementary Table 9 for a complete list of *P* values. **a**, Haploinsufficient genes; **b**, genes in GWAS catalogue; **c**, disease genes in ClinVar; **d**, housekeeping genes. **e**–**h**, log_2_ odds ratios for each cell type shown for genes identified as haploinsufficient (**e**), gene in GWAS catalogue (**f**), disease genes in ClinVar (**g**) or housekeeping genes (**h**) and having at least a given number of loops ending at its promoter (*adjusted *P* < 0.05 by two-sided Fisher’s exact test). See Supplementary Table 9 for a complete list of enrichments and *P* values.

We next investigated whether the number of physically interacting enhancers could be related to the basic properties of a gene. We hypothesized that genes that encode products with effects that depend strongly on their levels of expression (‘dosage-sensitive’ genes) would have more enhancer contacts to support a more robust regulatory architecture than other genes. To test this idea, we obtained a list of genes that were annotated as haploinsufficient (such that loss of one copy leads to pathogenicity)^45^. Haploinsufficient genes were enriched among genes with a higher number of loops, enhancers, and cell-type-specific enhancers connected to them (Fig. 3g, Extended Data Fig. 4a, e, Supplementary Table 9), suggesting a link between the dosage pathogenicity of a gene and increased regulatory contacts. We next tested whether other categories of human disease-related genes also tended to have more distal contacts. Analogous to the case for haploinsufficient genes, we find that genes identified as being disease-associated in GWASs^46^ tended to be more highly connected to distal regions, including enhancers (Extended Data Fig. 4b, f). The same was true for genes with a reported pathogenic or likely pathogenic variant in ClinVar^47^ (Fig. 3h, Extended Data Fig. 4c, g), indicating that genes associated with both common and rare human diseases possess more extensive regulatory wiring than other genes. By contrast, housekeeping genes, which we defined as being broadly expressed among our 24 cell types (Methods), were depleted from genes with higher numbers of loops (Fig. 3i, Extended Data Fig. 4d, h). This is consistent with the finding that housekeeping genes active during mouse development have a median of zero enhancers^48^. Together, these results indicate that genes for which misregulation makes an organism particularly vulnerable have a complex regulatory architecture that may ensure correct expression through the redundancy or fine-tuning of regulatory interactions.

## Loop interaction frequency and gene expression

**Fig. 4:**
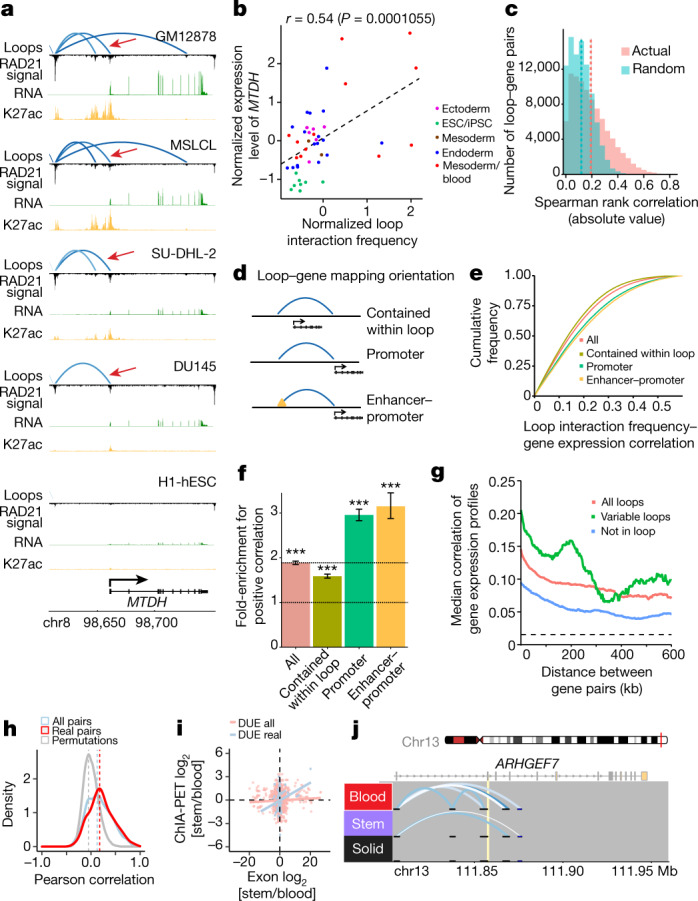
Variable chromatin loops correspond to changes in gene expression levels and alternative splicing. **a**, Example of chromatin loop changes with accompanying changes in gene expression and chromatin activity. Red arrow indicates a loop that links an active H3K27ac site to the promoter of *MTDH*. **b**, Pearson correlation between loop interaction frequencies and expression levels for the enhancer loop and *MTDH* (red arrow in **a**); sample size, 46 (23 cell types × 2 replicates). **c**, Spearman rank correlation (absolute value) between loop interaction frequency and gene expression levels for all loop–gene pairs versus randomized loop-gene pairs (*n* = 90,657, *P* < 2.2 × 10^−16^, two-sided Wilcoxon rank-sum test). **d**, Schematic of various ways to map loops to genes. **e**, Spearman rank correlation (absolute value) between loop interaction frequency and gene expression for different groups of loop–gene pairs. **f**, Fold-enrichment for positive correlation between loop frequency and gene expression levels for different groups of loop–gene pairs (all, *n* = 90,655 pairs; promoter, *n* = 18,628; contained, *n* = 40,719; enhancer–promoter, *n* = 4,421). Odds ratio ± 95% confidence interval (CI); ***P < 2.2 × 10^−16^; two-sided Fisher’s exact test. **g**, Median Spearman rank correlation of expression levels between distance-matched pairs of genes that are not located in the same loop (blue), are located in the same loop (red), or are located in the same variable loop (green). **h**, Pearson correlation of the normalized ChIA-PET anchor counts and the exon counts across all cell types for exon–loop pairs (red, *n* = 277), exon–loops pairs of the same gene (blue, *n* = 1,347) and 100 permutations of the exon associated to the anchor (grey, *n* = 27,700). Red versus blue, *P* = 9.2 × 10^−3^; red versus grey, *P* = 8.90 × 10^−193^). **i**, Scatterplots of the DUE and anchor counts for real pairs (blue, *n* = 111) and other exon–loop pairs within the same gene (pink, *n* = 1,347). **j**, Example of an intragenic loop that affects exon inclusion for gene *ARHGEF7*. Exon 6 (yellow) is included in the blood-specific group, but not in the stem-like/embryonic or solid groups.

We next investigated the extent to which changes in loop interaction frequency corresponded to changes in gene expression. For example, we observed physical interaction between a distal enhancer and the promoter region of the gene *MTDH*, a known oncogene that activates the NFκB pathway^49^. This interaction was frequent in blood cell lines (for example, GM12878, MSLCL, and SU-DHL-2), and accompanied by higher expression of *MTDH* RNA. Conversely, in cell types where looping with the promoter region was reduced or absent (for example, H1-hESC), we observed less expression (Fig. 4a, b). Globally, loop interaction frequencies were significantly, but only modestly, correlated with gene expression levels (Fig. 4c). We observed that this correlation grew slightly stronger when examining loops that connect a promoter to an enhancer element (Fig. 4d, e, Methods). Moreover, the correlation between loop frequency and gene expression tended to be more positive in these cases (Fig. 4e, f, Methods), which is consistent with a model in which a direct connection between promoter and enhancer drives gene expression^12^.

Loops frequently contain more than one gene, which could facilitate the co-regulation and co-expression of gene pairs^9,10,50^. We tested whether pairs of genes located within the same loop showed more correlated expression across cell types than those not contained in the same loop. We found a higher correlation among genes that shared a loop, which decreased as the distance between genes increased (Fig. 4g), perhaps because of a reduction in sharing of local regulatory elements.

## Chromatin loops regulate alternative splicing

**Extended Data Fig. 5:**
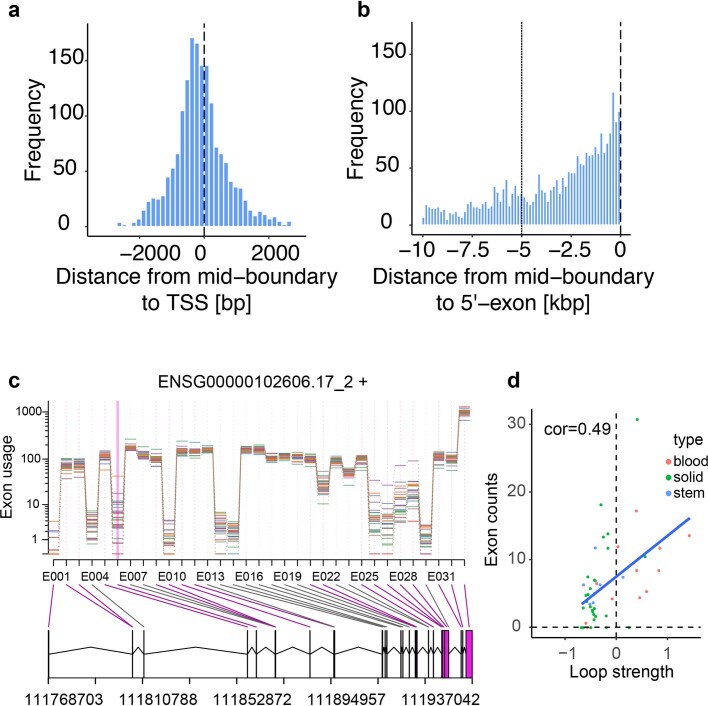
Chromatin loops are associated with alternative splicing across cell types. **a**, **b**, Distribution of the distance (bp) between the centre of the loop anchors and the TSS (**a**) or the exon 5′ boundary (**b**). **c**, DEXSeq plot showing the differential exon usage of all exons for gene *ARHGEF7*, highlighting exon 6, which is affected by an intragenic loop in the blood cell types. **d**, Scatterplot of the normalized counts of exon 6 in *ARHGEF7* with respect to the log_2_-transformed fold change in loop strength for all cell types (*n* = 44 (22 cell types × 2 biological replicates); Pearson correlation, 0.49).

A recent report^51^ found a link between intragenic CTCF-mediated chromatin loops and alternative splicing within the same cell type across individuals. To assess whether a similar mechanism might drive cell-type-specific isoform use, we identified 1,372 loops associated with 1,074 genes that linked the promoter and the gene body (Extended Data Fig. 5a, b). Loop strength showed the highest correlation with differentially used exons (DUEs) (Methods) that were next to the loop anchor when comparing the normalized signal across all cell types (Fig. 4h) and also when using fold change for a specific lineage (Fig. 4i). These results suggest that the presence of an intragenic loop can affect the inclusion of the exon next to it, as exemplified by *ARHGEF7*, which selectively includes exon 6 in cell types where the loop is present (Fig. 4j). Consequently, we observed a high correlation between loop strength and exon abundance (*R* = 0.49) (Extended Data Fig. 5c, d).

## Group-specific loops

Clustering of interaction frequencies across the genome revealed three distinct cell type clusters—blood, embryonic, and solid-tissue-derived (Fig. 2b). We next sought to identify and characterize loops that were present in each group. We rank-ordered all loops tested for variability according to the extent to which their interaction frequency was elevated in cell types in one group compared to the other two (Methods). We hypothesized that group-specific loops were relevant to the determination and maintenance of cell fate, which are governed by cell-type-specific TFs acting on proximal and distal regulatory elements. To examine enrichment of TFs at loop ends, we intersected each set of group-specific loops with motif positions for 598 TFs^52,53^.

**Fig. 5:**
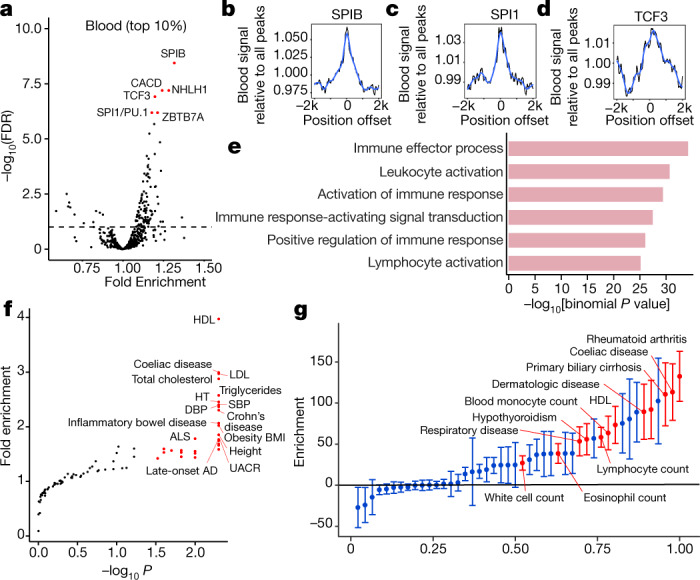
Characterization of group-specific loops. **a**, Fold-enrichment of 598 TF motifs in blood-specific chromatin loop ends (*n* = 3,384). Significance assessed using two-sided Fisher’s exact test with Benjamini–Hochberg correction for multiple hypothesis testing. Top hits are highlighted in red; complete enrichment results are provided in Supplementary Table 9. **b**–**d**, Chromatin accessibility determined by ATAC–seq at blood-specific loop anchors centred at the motif instances for SPIB, SPI and TCF3. **e**, Biological processes associated with blood-specific chromatin loops (*n* = 3,384). Enrichment was assessed using the GREAT^66^ tool. **f**, Enrichment of disease-specific GWAS SNPs (*n* = 86 diseases) in blood-specific loop ends (*n* = 3,384) assessed by a *P* value permutation test. HDL, HDL cholesterol; LDL, LDL cholesterol; HT, hypertension; SBP, systolic blood pressure; DBP, diastolic blood pressure; BMI, body mass index; UACR, urinary albumin–creatinine ratio; ALS, amyotrophic lateral sclerosis; AD, Alzheimer’s disease. **g**, Association of blood-specific chromatin loop anchors (*n* = 3,384) with GWAS traits observed by partitioned LD score regression^62^ using a common set of 47 traits^63^ (*n* = 1,100,000 HapMap3 SNPs, block jackknife *t*-test; mean ± s.d.).

**Extended Data Fig. 6:**
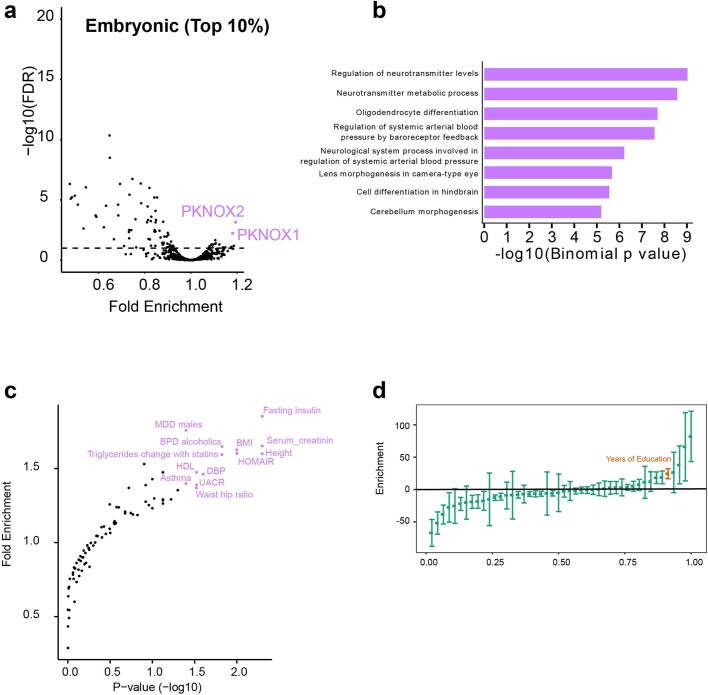
Transcription factor analysis, GO enrichments and GWAS for embryonic-specific loops. **a**, Fold-enrichments of 598 TF motifs in chromatin loop ends that are embryonic-specific (*n* = 2,894). Significance was assessed using two-sided Fisher’s exact test. *P* values were adjusted for multiple hypothesis testing using the Benjamini–Hochberg procedure. **b**, Biological processes associated with embryonic-specific chromatin loops (*n* = 2,894). Enrichment was performed using GREAT^66^. **c**, Enrichment of disease-specific GWAS SNPs in embryonic-specific loop ends. MDD, major depressive disorder; BPD, bipolar disorder; HOMAIR, homeostatic model assessment for insulin resistance. **d**, Association of embryonic-specific chromatin loop anchors (*n* = 2,894) with GWAS traits observed by partitioned LDSC^62^ using a common set of 47 traits^63^; (*n* = 1,100,000 HapMap3 SNPs, block jackknife *t*-test, mean ± s.d.).

Among the most significantly enriched TFs at blood-specific loop anchors were haematopoietic TFs involved in lymphoid B- and T-cell development, such as SPIB^54^, SPI1/PU.1^55^, TCF3 (which is mutated in most Burkitt lymphomas^56^), and ZBTB7A (which is recurrently mutated in acute myeloid leukaemia^57^) (FDR < 5%) (Fig. 5a, Methods). At the embryonic-specific loop anchors, we found modest enrichment for a smaller set of TFs, among them PKNOX1 (which regulates haematopoietic stem and progenitor cell activity^58^) and PKNOX2 (which is essential for limb development^59^) (FDR < 5%) (Extended Data Fig. 6a). Our results were fairly robust to the choice of threshold used to define group-specific loops (Methods).

To confirm the motif analysis, we reasoned that functional motifs are more likely to be present in open chromatin and thus we expected to see enrichment of chromatin accessibility signal. Indeed, the motif instances for the haematopoietic TFs SPIB, SPI1/PU.1, and TCF3 coincided with higher chromatin accessibility at blood-specific loop anchors than in the all-loops, as measured by assay for transposase-accessible chromatin using sequencing (ATAC–seq) in the blood lineage cells (Fig. 5b–d, Methods). This supports the notion that haematopoietic TFs are probably more active in blood-specific loops.

Next, we investigated whether cell-type-specific loops are associated with cell-type-specific biological processes, which would indicate that cohesin loops are integral to cell-type-specific transcriptional programs. Loops that were present more frequently in blood cell types than in other cell types were enriched for genes involved in leukocyte activation (*P* < 10^−31^), mature B cell activation (*P* < 10^−20^), and numerous other immune-related categories (Fig. 5e, Supplementary Table 6, Methods). Loops that were gained in the embryonic group were enriched for genes with a more complex pattern of functional categories, including differentiation and morphogenesis (Extended Data Fig. 6b). These results suggest that the cell-type-specific differences in chromatin looping are likely to be functionally important^60^.

Finally, we investigated to what extent disease-associated variants identified by GWASs tend to occur at loop ends. To this end, we intersected GWAS single-nucleotide polymorphisms (SNPs) from 86 traits^61^ with each set of group-specific loop ends (Methods). Similar to the GO enrichment analysis, we identified enrichment of GWAS SNPs for different sets of diseases in each set of group-specific loops (Fig. 5f, Extended Data Fig. 6c). The blood-specific loops were enriched for SNPs associated with autoimmune diseases, including multiple sclerosis, coeliac disease, and Crohn’s disease (Fig. 5f). Lipid-associated traits (for example, LDL, HDL, and total cholesterol) were also enriched. These enrichments were not significant in the embryonic-specific loops (Extended Data Fig. 6c); we confirmed this result through a relative enrichment test, which directly compared each set of group-specific loops (Supplementary Table 7, Methods). The most significantly associated traits identified for embryonic-specific loops were fasting insulin, serum creatinine, and height (Extended Data Fig. 6c), the latter of which has previously been associated with stem-cell-specific chromatin patterns^39^.

**Extended Data Fig. 7:**
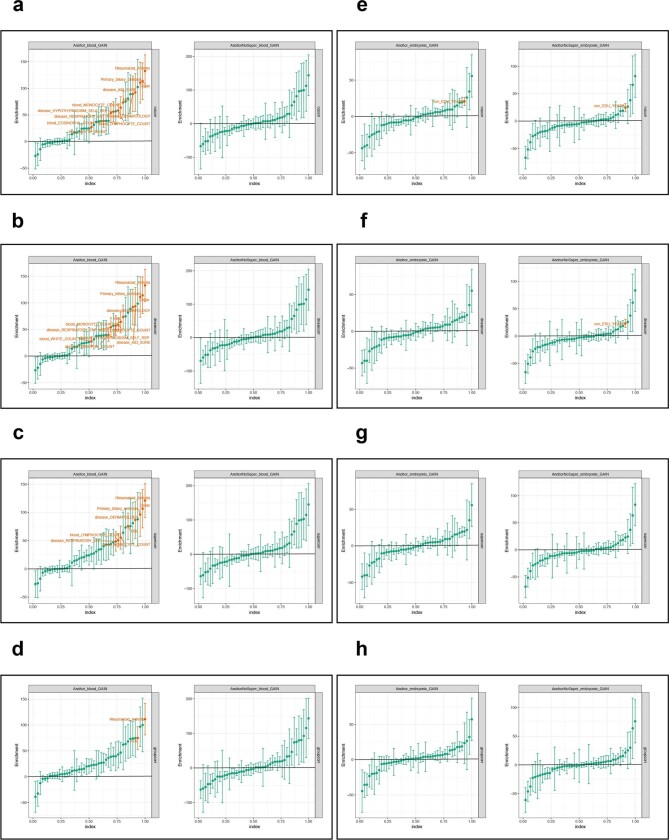
Association of chromatin loops with GWAS traits. **a**–**h**, Association of blood-specific (**a**–**d**) and embryonic-specific (**e**–**h**) chromatin loop anchors with GWAS traits observed by partitioned LDSC^62^ using a common set of 47 traits^63^. (*n* = 1,100,000 HapMap3 SNPs, block jackknife *t*-test, centre values indicate the mean ± s.d.). Within each panel: left, all blood-specific loops (**a**–**d**) or embryonic-specific loops (**e**–**h**); right, set of loops that does not overlap with super enhancers. All panels adjusted for the set of baseline line traits as previously described^62^; in addition, **b**, **f** are adjusted for all RAD21 loops; **c**, **g** are adjusted for super-enhancers across all cell types, within blood-specific and embryonic–specific loops; **d**, **h** are adjusted for cell-group-specific signal^62^ and global Roadmap annotation^67^.

We confirmed the GWAS enrichments we observed using GRASP overlaps by a complementary approach, partitioned linkage disequilibrium (LD) score regression^62^, using a common set of 47 traits^63^. Similar traits were enriched in the blood-specific group of loop anchors, including HDL cholesterol and autoimmune-related diseases, along with a number of blood cell traits (Fig. 5g). In embryonic lineages, the only significantly enriched trait at FDR = 1% was ‘years of education’, which is thought to be driven by brain associations and was also consistent with the embryonic and neural progenitor populations in this group^63^ (Extended Data Fig. 6d). We also examined the sensitivity of the LD score regression approach to additional corrections for underlying genomic features, for example, super enhancer annotations, and observed that the overall trends of enrichment remained consistent (Extended Data Fig. 7, Supplementary Table 8, Methods).

Together, our results suggest that distinct sets of TFs may help to facilitate cell-type-specific loops, which in turn contain functionally related genes that are critical to the function of each particular cell type^60^, emphasizing the important role of chromatin loops in human traits and suggesting that analysis of the effects of genetic variants in these regions may provide mechanistic insights into disease.

## Discussion

We have generated one of the most comprehensive 3D chromatin interaction data sets to date, spanning 24 cell types. Owing to the high reproducibility of our data, we were able to identify loops whose interaction frequencies varied across our panel of cell types. About 28% of loops genome-wide varied significantly among cell types and were associated with cell-specific differences in gene expression. The differences in gene expression associated with loop variation are relatively modest, perhaps suggesting that not all varying chromatin loops have functional consequences or that a subset of variable loops may be poised to alter gene expression in specific developmental or physiological contexts. Notably, we found that neighbouring genes, which tend to be on average more co-expressed than non-neighbouring genes, showed more strongly correlated gene expression when contained within the same chromatin loop than when they were located next to each other but did not share a loop. Together, these results indicate that chromatin looping has a role in regulating gene expression, and point to the ability of loops to coordinate the expression of functionally related sets of genes, such as pathways or protein complexes^60^.

The diverse cell types we studied clustered into three main groups—blood, embryonic, and derived from solid tissues—based on shared commonalities in cohesin-mediated chromatin looping. Analyses from both the GTEx Consortium^33^ and Roadmap Epigenomics Project^39^—which profiled dozens of tissues and cell types for gene expression and histone modifications, respectively—showed very similar grouping, indicating that both blood and embryonic cell types are likely to have gene regulation programs that differ strongly from those of solid tissues. To our knowledge, this is the first time that quantitative measurements of cohesin-mediated chromatin loops have also been shown to correspond to cell-type identity.

Our results build on previous ENCODE work that has shown that gene architecture is highly variable throughout the human genome. Notably, we have shown that the extent of long-range contacts of a gene correlates with its function and role in human disease. Genes with few contacts are enriched in housekeeping genes, which could reflect simpler circuitry for constitutive, steady expression. Highly connected genes are more strongly associated with both common and rare classes of human diseases, as demonstrated by their enrichment in ClinVar^47^ and GWAS genes^46^ and in genes that cause disease when haploinsufficient^45^. These observations may indicate that one function of a more extensive regulatory architecture is to safeguard the expression of dosage-sensitive genes.

Maps of 3D chromatin interactions have become increasingly useful in explaining how distal regulatory elements can exert their influence. Here, we have demonstrated how knowledge of cell-type-specific interactions can further expand the utility of such maps. For example, we found that GWAS SNPs were enriched in loops observed in cell types that have been shown to be relevant to a particular disease. These findings suggest that our data set could have multiple future applications. GWAS and cancer genomics studies continue to deposit disease-related sequence variations into public databases, and most of these variants fall into non-coding regions. As we have demonstrated here, intersecting these variants with cell-type-specific chromatin loops may help to explain how such sequence variation leads to disease.

## Methods

### Cell lines

The cell types and lines in this study were either obtained from cell repositories or established or differentiated in the Snyder and Dalton laboratories at Stanford University and the University of Georgia, respectively (Supplementary Tables 1, 2). All tissue culture was done according to the manufacturer’s recommendations. One of the commercially available cell lines, K1 (thyroid, papillary carcinoma), is on the list of commonly misidentified cell lines (ICLAC). The relevant cell line (CVCL_9918) was also derived from a thyroid papillary carcinoma. In the event of misidentification, the conclusions of our study would not be affected because both cell lines represent papillary thyroid carcinoma.

### ChIA-PET experiments

We performed ChIA-PET experiments with modifications to previously published protocols^2,22^. These modifications have also been independently described^17,23^. We used Illumina’s Nextera tagmentation to generate sequencing libraries. In brief, cells were crosslinked and subjected to nuclear lysis followed by chromatin shearing (no restriction enzyme was used). Immunoprecipitation was performed overnight at 4 °C with antibodies against the cohesin subunit RAD21 (Abcam Anti-RAD21 antibody (ab992) https://www.encodeproject.org/antibodies/ENCAB529YRC/). The immuno-complexes were pulled down with Protein-G dynabeads (Life Technologies #10003D, New York). Biotinylated linkers were ligated to the enriched fragments, followed by proximity ligation overnight at 16 °C.

Crosslinking was reversed at 65 °C with the use of Proteinase K followed by DNA purification. We used Illumina Nextera Transposase to add sequencing adapters to ChIA-PET libraries. Biotinylated fragments were enriched by pull-down with Streptavidin Dynabeads (M-280; Lifetechnologies #11205D, New York). The final libraries were sequenced on an Illumina HiSeq 2000.

### ChIP–seq experiments

Chromatin immunoprecipitation followed by massively parallel sequencing was carried out as previously described^32^. Cells were crosslinked with formaldehyde at a final concentration of 1% for 10 min at room temperature. The reaction was quenched with glycine at a final concentration of 125 mM and nuclear lysates were sonicated using a Branson 250 Sonifier (power setting 2, 100% duty cycle for 7 × 30-s intervals). Clarified lysates corresponding to 20 million cells were treated with 1–5 μg of antibody against H3K27ac (Abcam #4729; https://www.encodeproject.org/antibodies/ENCAB000BSK/) coupled to Protein G Dynabeads (Life Technologies #10003D). The protein–DNA complexes were washed with RIPA buffer and eluted in 1% SDS TE at 65 °C. Following cross-link reversal and purification, the ChIP DNA sequencing libraries were generated according to Illumina DNA TruSeq DNA Sample Preparation Kit Instructions (Illumina Part # FC-121-2001). Pooled libraries were sequenced on an Illumina Hi-Seq 4000. To generate high-quality data sets, we used the same antibodies as in our previous studies^9,32^ which have been validated according to ENCODE standards^27^.

### RNA-seq experiments

RNA samples were extracted using the Qiagen All-Prep kit, following the manufacturer’s instructions. Libraries were prepared from total RNA using the TruSeq Stranded Total RNA Library Prep Kit, following the manufacturer’s instructions. All libraries were sequenced on the Illumina Hiseq 4000

### ATAC–seq experiments

ATAC–seq was carried out as previously described^68^ and sequencing was carried out on an Illumina HiSeq 2000 with 2 × 100 paired-end sequencing.

### ChIA-PET processing pipeline

ChIA-PET data were generated in replicate for all 24 cell lines; all libraries were sequenced to an average depth of 214 ± 5.5 (mean ± s.d.) million paired-end reads (referred to as paired-end tags or PETs) (Supplementary Table 2). Data were processed in a similar way to the workflow used in the Mango toolkit^28^, as follows.

#### Trim adaptor sequences

Illumina Nextera adaptor sequences (CTGTCTCTTATA and TATAAGAGACAG) were trimmed from all PETs using cutadapt in paired-end mode (version 1.11; non-default parameters: -q 15 -O 4 -m 20).

#### Trim linker sequence

All PETs were scanned to identify and remove the linker sequence (GTTGGATAAG), as well as any sequences downstream of the linker sequence. PETs less than 20 bp in length after linker removal were discarded.

#### Align paired-end sequences

Each set of paired-end reads was aligned to the hg19 genome separately using bowtie (version 0.12.8; non-default parameters: -n 2 -l 50 -k 1 --mapq 40 --best -m 1). Paired-end reads that mapped to multiple locations were discarded.

#### Remove duplicate paired-end sequences

PETs that mapped to identical locations were filtered to retain only a single PET.

#### Generate a set of unified peak calls

For each sample, the two sets of uniquely mapped paired-end reads were merged and peaks were called using MACS2^69^ (version 2.1.1.20160309; parameters: -g hs -f BED -q 0.01). Peak calls across all samples were combined and then extended by 500 bp in either direction. Overlapping peaks were merged to form a single interval that spanned all overlapping peaks, after which peaks in ENCODE-defined blacklist regions were filtered. In total, we obtained 286,620 RAD21 peaks (Supplementary Table 3). These merged peak regions were used as our ‘anchor regions’ for all subsequent analysis.

#### Generate a set of linked paired peaks

For all pairs of peaks that were >10,000 bp and <5,000,000 bp apart on chr1-22 and chrX, the total number of PETs that linked each pair was tabulated. For samples with >2,250,000 unique PETs, the total number of PETs was down-sampled to 2,250,00 before any further analysis.

Our final data set consisted of a matrix, **M**_*i*,*j*_, in which each row (*i*) represents a single paired-peak, and each column (*j*) represents a single sample. Element *m*_*i,j*_ indicates the number of PETs linking the two anchor regions. We normalized the data by standardizing each row in **M**_*i,j*_, and then quantile-normalizing the columns. The range of values in each column was then re-scaled to between 0 and 1000.

### Generating the pan-cell line loop-call data set

Unique PET data (that is, data from ‘Remove duplicate paired-end sequences’ in the ChIA-PET processing pipeline above) from all cell lines and all replicates were pooled together. Next, we tabulated the number of PETs that connected all pairs of anchor regions >10 kb and <5 Mb apart in our unified peak set (Supplementary Table 3). Finally, the Mango scoring methodology^28^ was used to assign each peak pair a *P* value; Mango uses a Bayesian scoring methodology to determine the expected number of PETs connecting any two regions on the basis of the distance between the two regions and the local ChIP-efficiency. We used a threshold of *P* < 2.3 × 10^−9^ to arrive at our pan-cell line loop set (Supplementary Table 4). We used a relatively stringent cutoff due to the large number of PETs being analysed. At this cutoff our FDR was 2.7 × 10^−6^ using the Benjamini–Hochberg procedure and 0.013 using the Bonferroni approach. For all subsequent analysis described below, we used the FDR estimate from the Benjamini–Hochberg procedure.

### RNA-seq processing

RNA-seq data were generated in replicate for 23 out of 24 cell lines (Supplementary Table 2); we obtained on average 66 ± 18 million paired-end reads per sample (mean ± s.d.). For samples with >60 million reads, FASTQ files were down-sampled to 60 million reads before further analysis. Transcript abundances were quantified using kallisto^70^ (version 0.43.0; non-default parameters:–bias). Transcript sequences (that is, target sequences) were obtained from Gencode (release 25; lifted to GRCh37 coordinates). Duplicate transcripts were removed, as well as transcripts not classified as ‘protein_coding’ or ‘lncRNA’, yielding a final list of 93,430 transcripts. For all analyses, we considered only 69,598 transcripts with a maximum abundance of >1 transcripts per million (TPM) across all 23 cell lines. To produce gene-level estimates of expression, we summed the TPM values for all transcripts that belonged to the same gene. For all analyses, we considered only 22,197 genes with a maximum abundance of >1 TPM across all 23 cell lines. For GM12878 cells, we used data from a previous study^32^. To normalize RNA-seq data, we first standardized (that is, *z*-score scaled) TPM values for each transcript or gene across all cell lines and then quantile-normalized all transcript or gene abundance levels between samples.

To visualize RNA-seq data as signal tracks, down-sampled FASTQ files were aligned to the hg19 genome using HiSat2 (version 2.0.5; non-default parameters: -X 1000–fr–no-mixed–no-discordant)^71^, after which genome-wide coverage tracks were produced using bedtools (bedtools genomecov -bga -split -ibam). Coverage values were scaled by a constant factor (10^9^/total number of reads) to account for differences in sequencing depth.

### H3K27ac ChIP–seq data processing

ChIP–seq data were generated in replicate for 22 out of 24 cell lines (Supplementary Table 2); we obtained on average 43 ± 9 million paired-end reads per sample (mean ± s.d.). Illumina TruSeq adaptor sequences were trimmed using cutadapt in paired-end mode (non-default parameters: -q 15 -O 4 -m 20). Reads were aligned to hg19 using bowtie (version 0.12.8; non-default parameters: -m 1–fr–chunkmbs 500 -n 2 -l 50–mapq 40 –best) after which duplicate reads were removed using Picard MarkDuplicates. Finally, peaks were called using MACS2^69^ (non-default parameters: -q 0.01). Peaks across all samples were combined and overlapping peaks were merged to form a single interval spanning all overlapping peaks. Peaks seen in fewer than two samples, peaks that overlapped ENCODE blacklisted regions (https://sites.google.com/site/anshulkundaje/projects/blacklists), and peaks on chrM and chrY were removed from further consideration. The final list of ‘enhancer’ regions consists of 288,711 peaks (Supplementary Table 5).

Genome-wide signal tracks for each sample were generated in two stages: (i) assess ChIP–seq quality and obtain the predominant fragment length using phantompeakqualtools (https://code.google.com/archive/p/phantompeakqualtools/); (ii) use align2rawsignal (https://code.google.com/archive/p/align2rawsignal/wikis/Method.wiki) to generate signal track (parameters: -*n* = 5, -k = epanechnikov, -l = [fragment length from step (i)], -w = 150, -f = 0). Finally, for each cell line, we extracted the signal in each of 288,711 peaks using bwtools^72^ (bwtools extract bed) and calculated the average value for each peak. The final data set consists of a matrix **M**_*i*,*j*_, in which each row (*i*) represents a single peak and each column (*j*) represents a single sample. We normalized the data by standardizing each row in **M**_*i*,*j*_, and then quantile-normalizing the columns. These normalized data were used for all downstream analyses.

### Identifying super-enhancers

To call super-enhancers in each cell line we used the ROSE pipeline^73,74^ (default parameters).

### ATAC–seq data processing

ATAC–seq data were generated in 18/24 cell lines; we obtained on average 13 ± 7 million paired-end reads. Adaptor sequences were trimmed using cutadapt in paired-end mode (non-default parameters: -q 15 -O 5 -m 30). Reads were aligned to hg19 using bowtie (version 0.12.8; non-default parameters: -X 2000, -m 1) after which duplicate reads were removed using Picard MarkDuplicates. Genome-wide signal tracks for each sample were generated using align2rawsignal (https://code.google.com/archive/p/align2rawsignal/wikis/Method.wiki)

### Overlap between cohesion-mediated chromatin loops and high-resolution Hi-C loops, contact domains and TADs

We obtained the coordinates for Hi-C loops from seven cell lines (including GM12878) and contact domains in GM12878^12^ to calculate the overlap with our pan-cell line loops (Fig. 1d). We also obtained the coordinates for TADs across 21 human tissues and cell types^19^ and compared the size of these TADs to our pan-cell line loops (Fig. 1c).

### Assessing CTCF motif orientation

A list of CTCF motif positions and orientations was downloaded from the ENCODE project^53^. We used the CTCF_known1 motif for all analysis; this motif most closely matched the one used in a previous analysis^12^. Next, for all loops that contained exactly one instance of the CTCF motif at both ends (that is, in both anchor regions), we calculated the percentage of loops that had each of four possible orientations (+/−, −/+, +/+, and −/−). This result was relatively robust to the choice of threshold used to define the pan-cell line loop set (FDR<10^−5^: 69%, FDR<10^−4^: 68%, FDR<0.01: 66%, FDR<0.05: 64%).

### Characterizing ‘hub’ anchor regions

Promoter regions were defined as a 500-bp region immediately upstream of a gene; gene coordinates were taken from Gencode Release 25. Enhancer regions were defined as the set of 288,711 H3K27ac peaks defined from our ChIP-seq data set (see ‘H3K27ac ChIP-seq data processing’ for more information). All anchor regions were binned by the number of interactions they had in the ‘merged loop-call’ data set (Supplementary Table 4). We assessed whether anchor regions in a particular bin were enriched for overlap with functional elements such as enhancers, promoters, or contact domain boundaries (taken from a previous publication^12^) using Fisher’s exact test. For each bin, we tabulated the number of anchor regions that overlapped or did not overlap a functional element; we then tabulated the number of anchor regions in all other bins that overlapped or did not overlap a given functional element. These four values were used to populate a 2 × 2 contingency table and to compute a significance of enrichment. To test the robustness of our results with respect to the threshold used to define the set of merged loop-calls, we repeated this analysis using an FDR<1% (summary statistics for the fold-enrichment and *P* values can be found in Supplementary Table 9).

Qualitatively, we observe very similar results to Fig. 3a—regions with many interactions are enriched for enhancers and contact domain boundaries, whereas promoters tend to overlap regions with fewer interactions.

### PCA

We performed PCA on the matrix of normalized interaction frequencies of 85,294 loops by 48 samples using the prcomp function in R (default options). The 85,294 loops were derived from the set of pan-cell line loops (Supplementary Table 4) after filtering for interactions that had >4 PETs in at least one sample. We repeated the analysis using the entire set of pan-cell line loops at various FDR cutoffs and observed high correlation in PC1 and PC2 values (FDR < 10^−5^: *r*_PC1 _= 0.996, *r*_PC2 _= 0.995; FDR < 0.05: *r*_PC1 _= 0.983, *r*_PC2 _= 0.981). We also observed similar results when using different PET cutoffs to filter loops (>2 PETs: *r*_PC1 _= 0.999, *r*_PC2 _= 0.997; >10 PETs: *r*_PC1 _= 0.993, *r*_PC2 _= 0.985).

### Testing for similarity in interaction profiles between similar cell types

For a pair of samples, we calculated the Spearman rank correlation between the raw PET counts across the set of pan-cell line loops identified (124,830 loops) for which there were at least four PETs in at least one sample (85,294 loops). For Fig. 2c, we plotted the distribution of correlation coefficients for the following groups: ‘all’ (all pairs of samples excluding replicates); ‘same germline layer’ (the assignment of individual cell lines to germline layers is provided in Supplementary Table 1; note that replicate pairs are included in this grouping); ‘same tissue’ (the assignment of individual cell lines to tissue is provided in Supplementary Table 1; note that replicate pairs are included in this grouping); ‘biological replicates’ (replicate samples); and ‘isogenic cell types’ (these include cell lines derived from a single male individual (MSLCL, MSFIB, and MSiPS); note that replicate pairs are included in this grouping).

Differences in the distribution of correlation coefficients were assessed using a two-sided Wilcoxon rank-sum test. *P* values were corrected for multiple hypothesis testing using the Bonferroni approach. We repeated the analysis including replicate pairs in the ‘all’ distribution and observed similar results (*P*_all vs isogenic cell types_ = 0.4, *P*_all vs biological replicates_ = 4.38 × 10^−15^, *P*_all vs same tissue_ = 2.52 × 10^−38^, *P*_all vs same germline layer_ = 1.23 × 10^−25^). The results were also robust to the particular PET threshold used (we examined thresholds of 1–20 PETs in at least one sample; Extended Fig. 2e). Finally, qualitatively similar results were observed when we used normalized PET interaction frequencies instead of raw PET counts (*P*_all vs isogenic cell types_ = 0.79, *P*_all vs biological replicates_ = 2.6 × 10^−15^, *P*_all vs same tissue_ = 9.2 × 10^−27^, *P*_all vs same germline layer_ = 3.2 × 10^−9^).

### Assessing the effect of technical confounders on loop interaction frequency

For each ChIA-PET sample, we recorded the following potential confounding variables: batch (the set of samples which were processed at the same time and pooled together for sequencing); normalized strand cross-correlation coefficient (NSC; a metric of ChIP efficiency/quality^27^); number of peaks called; and number of uniquely mapped PETs between 10 kb and 5 Mb.

We tested for an association between principal components 1–10 (see ‘PCA’) and each covariate described above using a linear model (PC ~ technical_variable) and assessed significance using the ANOVA implementation in R. *P* values were corrected for multiple hypothesis testing using the Benjamini–Hochberg procedure. At an FDR <10%, we detected no significant associations. Thus, we chose not to correct for any of these technical confounders when testing for variable loops (see below).

### Identifying variable loops

We began with the set of 124,830 merged loop calls and filtered loops to include only those that had ≥4 PETs in at least one sample yielding 85,294 loops. Next, we estimated the mean to variance relationship in the data using the voom method^75^ and used the inverse variance weights in the subsequent analysis. To assess loops that exhibited significant variability across cell types, while accounting for technical variables observed between replicates from the same cell type, we used a linear mixed effects model as previously described^76^. For each of the 85,294 loops, we modelled the log(normalized interaction frequency) as a function of the cell line (treated as a random effect) using the ‘lmer’ function from the lme4 R package. We then compared the mixed effects model to a simple linear model that lacked the random effect component; a *P* value was then calculated using a log-likelihood ratio test. *P* values were corrected for multiple hypothesis testing using the Benjamini–Hochberg procedure.

We tested two alternate approaches and found significant overlap with the approach described above.

#### Linear model

For each loop, we fitted a linear model [log(normalized interaction frequency) ~ cell type] and assessed its significance using the ANOVA implementation in R. *P* values were corrected for multiple hypothesis testing using the Benjamini–Hochberg procedure. At an FDR <10%, we found 21,353 loops; 20,926 of these were also found using the approach described above (98%; 2.34 fold-enriched compared to hypergeometric expectation)

#### Non-parametric approach

For each loop, we tested for differences in the normalized interaction frequency using a Kruskal–Wallis test. As a non-parametric approach is likely to be under-powered, we rank ordered all interactions according to *P* values and examined the overlap for the top 35,698 interactions (that is, the same number as found using the mixed effects linear model). A total of 23,117 overlapping hits were found (64% of the set found using the mixed effects linear model; 1.55 fold-enriched compared to hypergeometric expectation).

### Defining a set of non-variable loops (static loops)

To compare various attributes of our differential loops, we defined two sets of invariant or static loops as follows.

#### Static (null set) 1

For each of the 85,294 loops we tested for differential interaction frequency, we computed an ad hoc metric as follows:

$${{\rm{S}}{\rm{c}}{\rm{o}}{\rm{r}}{\rm{e}}}_{{\rm{s}}{\rm{t}}{\rm{a}}{\rm{t}}{\rm{i}}{\rm{c}}}=\frac{1}{{\rm{r}}{\rm{e}}{\rm{l}}{\rm{a}}{\rm{t}}{\rm{i}}{\rm{v}}{\rm{e}}\,{\rm{e}}{\rm{n}}{\rm{t}}{\rm{r}}{\rm{o}}{\rm{p}}{\rm{y}}}\times {\rm{m}}{\rm{e}}{\rm{a}}{\rm{n}}\,{\rm{P}}{\rm{E}}{\rm{T}}\,{\rm{f}}{\rm{r}}{\rm{e}}{\rm{q}}{\rm{u}}{\rm{e}}{\rm{n}}{\rm{c}}{\rm{y}}$$

in which relative entropy is defined as follows:

$${\rm{Relative}}\,{\rm{entropy}}=\sum _{j}{f}_{j}{\log }_{2}\frac{{f}_{j}}{{q}_{j}}$$

*j* sums across all samples (that is, cell lines) and *f*_*j*_ represents the fractional PET count in sample *j* (that is, the ratio of the number of PETs in sample *j* divided by the total number of PETs for this particular loop). *q*_*j*_ represents the fractional PET count under a null model assuming an equal number of PETs in each sample. In essence, a high static score would indicate a strongly interacting loop with uniform interaction frequencies across all cell lines. All loops were ranked in descending order by their static score and we selected the same number of high-scoring interactions as differential interactions identified (FDR <10%).

#### Static (null set) 2

From the set of 85,294 loops tested for differential activity, we selected a set of interactions found to not have differential activity (FDR >50%), but matched for the following properties to the set of differential interactions (FDR <10%): number of loops; distribution of loop sizes; and distribution of *P* values assigned by Mango (from the merged loop data set).

The last criterion helps to ensure that the static set of interactions is roughly comparable in quality to the differential interaction set.

### Defining housekeeping and cell-type-specific genes

For all 22,197 genes, we computed a relative entropy score as defined in ‘Defining a set of nonvariable loops (static loops)’ above. We then removed genes with low expression (minimum expression across all samples had to be >1 TPM). Genes in the top and bottom 10% as ranked by the relative entropy score were designated as ‘cell-type-specific’ and ‘housekeeping’ genes, respectively. Finally, we assessed whether variable or non-variable loops were enriched for housekeeping genes or cell-type-specific genes as follows. For the set of variable or non-variable loops (both null set 1 and null set 2), we tabulated the number that contained or overlapped more than one housekeeping or cell-type-specific gene. Similarly, we tabulated the number of variable or non-variable loops that contained or overlapped no genes in either the housekeeping or cell type-specific set. Enrichment was assessed using a two-sided Fisher’s exact test.

### Chromatin state analysis with cell-type-specific loop ends

Chromatin state calls using a 15-state model for 12 cell lines were obtained from the Roadmap Epigenomics Mapping Consortium^39^ (Supplementary Table 1). We merged chromatin states calls into eight categories as follows: (1) TSS: 1_TssA, 2_TssAFlnk; (2) BIV: 10_TssBiv, 11_BivFlnk; (3) TX: 3_TxFlnk, 4_Tx, 5_TxWk; (4) REPRESS: 13_ReprPC, 14_ReprPCWk; (5) REPEAT: 8_ZNF/Rpts; (6) ENH: 12_EnhBiv, 6_EnhG, 7_Enh; (7) HET: 9_Het; and (8) QUIES: 15_Quies.

 Next, for each cell line, we identified a set of loops that were present only in the cell line of interest (CellLine_query_) and not in all other cell lines (CellLine_others_) as follows: 1. Calculate a *t*-statistic based on the comparison of interaction frequencies (raw PET count) for all samples in CellLine_query_ and CellLine_others_. 2. Rank order each vector of *t*-statistics in descending order. 3. Define the set of cell-type-specific loops as the top 10% of loops identified in Step 2.

To assess the enrichment of various chromatin states at cell-type specific loop ends, we generated a 2 × 2 contingency table populated with the following four values: 1. Number of loop-ends that participated in a cell-type-specific interaction that overlapped a chromatin element. 2. Number of loop-ends that participated in a cell-type-specific interaction that did not overlap a particular chromatin element. 3. Number of loop-ends that did not participate in a cell-type-specific interaction that overlapped a particular chromatin element. 4. Number of loop-ends that did not participate in a cell-type-specific interaction that did not overlap a particular chromatin element.

Significance was assessed using the Fisher’s exact test. *P* values were corrected for multiple hypothesis testing (12 cell lines × 8 chromatin states) using the Benjamini–Hochberg procedure. We repeated our analysis using different rank thresholds to define the set of cell-type specific interactions by repeating this analysis using different thresholds (5%, and 15%) and assessed the robustness of our results, by comparing the overlap in enriched/under-enriched chromatin states. At a 5% rank threshold cutoff, eight cell lines had perfect agreement (H1-hESC, NCI-H1437, H9-hESC, HepG2, K562, LX, MSiPS, MSFIB). Three agreed for 7/8 chromatin states (HPAEC, GM12878, NP) and one agreed for only 5/8 (Jurkat). At a 15% rank threshold cutoff: Eight cell lines had perfect agreement (HPAEC, NCI-H1437, H9-hESC, HepG2, K562, LX, MSIPS, NP). Four agreed for 7/8 chromatin states (MSFIB, Jurkat, HepG2, and H1-hESC).

In cases of disagreement, except for H1-hESC, the typical change in result was the BIVALENT state going from over-enriched to no enrichment. For H1-hESC, the REPEAT state went from under-enriched to no-enrichment. Nevertheless, the vast majority of results were similar across all thresholds.

To assess whether cell-type-specific loops were enriched for TSS–TSS, TSS–ENH, or ENH–ENH, we first identified cell-type-specific loops, genes, and enhancer peaks as described above. To have adequate numbers, we defined the set of cell-type specific genes as the top 20% of genes identified using the procedure above.

Next, we counted the number of cell-type-specific loops whose ends overlapped one of the three chromatin state combinations described above. Similarly, we counted the number of non-cell-type-specific loops whose ends overlapped one of the three chromatin state combinations described above. An enrichment test was then performed using Fisher’s exact test.

### Testing for an association between gene expression level and number of linked enhancers

For each cell line, we identified a set of (i) cell-type specific loops (that is, high interaction frequency in cell line of interest and not in others), (ii) enhancers, and (iii) genes (that is, high normalized expression levels in cell line of interest and not in others) using the procedure outlined above (see ‘Chromatin state analysis with cell-type-specific loop ends’). Next, for each gene that was expressed in a single cell type of interest, we tabulated the number of cell-type-specific enhancers that were linked to its promoter. To generate Fig. 3f we aggregated results across all cell lines. To test for differences in the distribution of normalized expression levels between numbers of linked enhancers, we used the Wilcoxon rank-sum test. We repeated the analysis using different cutoffs to define cell-type specific loops, including 1% (*P*_1 vs 2 _= 0.008, *P*_1 vs 3+ _= 0.81, *P*_2 vs 3+ _= 0.37), and 15% (*P*_1 vs 2_ = 5.3 × 10^−11^, *P*_1 vs 3+ _= 2.5 × 10^−13^, *P*_2 vs 3+ _= 2.4 × 10^−3^). We also tested different cutoffs to define genes with cell-type-specific expression including 10% (*P*_1 vs 2 _= 1.4 × 10^−5^, *P*_1 vs 3+ _= 5.1 × 10^−4^, *P*_2 vs 3+ _= 0.47) and 17.5% (*P*_1 vs 2 _= 3.6 × 10^−9^, *P*_1 vs 3+ _= 2.7 × 10^−7^, *P*_2 vs 3+ _= 0.011). Lastly, we tested different cutoffs to define cell-type-specific enhancers including 5% (*P*_1 vs 2 _= 1.6 × 10^−8^, *P*_1 vs 3+ _= 3.1 × 10^−6^, *P*_2 vs 3+ _= 0.18) and 25% (*P*_1 vs 2 _= 1.6 × 10^−19^, *P*_1 vs 3+ _= 5.0 × 10^−13^, *P*_2 vs 3+ _= 0.049).

### Loop architecture in disease-associated genes

We downloaded the lists of disease-associated genes from ClinVar^47^, the GWAS catalogue^46^ and haploinsufficient genes^45^. The set of housekeeping genes was defined as above (‘Defining housekeeping and cell-type-specific genes’). For each list of genes, we tested the association of the gene being part of the specific category (ClinVar, GWAS or haploinsufficient) and having at least *X* loops connected to its promoter where *X* was a number from 1 to 10. We repeated the same test, filtering the loops for only enhancer loops (with a H3K27ac signal at the other end), and cell-type-specific enhancer loops (a H3K27ac mark in a given cell type). *P* values were calculated using Fisher’s exact test and corrected for multiple testing using the Benjamini–Hochberg approach. A list of all enrichments and *P* values is provided in Supplementary Table 9.

### Mapping genes to loops

To integrate gene expression and histone data, we generated a map of genes to loops as follows: ‘All’ (a gene was assigned to any loop within 1 kb of its start or end coordinates, as defined in Gencode version 25 lifted to hg19, or if the ORF overlapped partially with the loop); ‘Promoter’ (a gene was assigned to any loop for which its TSS was within 1 kb of either anchor region); ‘Contained’ (a gene was assigned to any loop it was entirely contained within (that is, start and end coordinates of the gene fell entirely within a loop) and its promoter was more than 1 kb from either anchor region); and ‘Promoter–enhancer’ (one loop end overlaps a promoter, the other end overlaps an H3K27ac peak).

### Linking gene expression changes to changes in loop interaction frequency

For each loop, we correlated the normalized interaction frequencies across all cell types (Spearman rank correlation; *n* = 23 cell types with RNA-seq and ChIA-PET data) with the normalized gene expression levels across all cell types. If a loop mapped to multiple genes, we computed all possible loop–gene correlations. As a control, we shuffled the mapping between loops and genes, while maintaining the total number of genes mapped to a single loop, and re-examined the correlation between loop interaction frequency and gene expression values. This procedure was repeated 100 times and we recorded the mean correlation coefficient for each loop–gene pairing.

In Fig. 4c, we have restricted our analysis to the set of variable loops (FDR < 10%) and plotted the distribution of actual versus randomized correlation coefficients (absolute value) for all loop-gene pairs (*n* = 90,657). We compared the distribution of actual correlation coefficients to ‘null’ correlation coefficients using the Mann-Whitney *U* test (*P* < 2.2 × 10^−16^). We repeated the analysis using the set of all loops tested for variable interaction frequencies (*n* = 251,678 loop-gene pairs) and observed significant results (*P* = 2.2 × 10^−16^), albeit with a lower mean correlation (0.17 versus 0.19 for the set of variable loops).

To assess what effect the mapping between loop and gene might have, we compared the distribution of correlation coefficients (absolute value) for all loop-gene pairings for all four maps described above (All, Promoter, Promoter–enhancer and Contained). Significance was assessed using a two-sided *t*-test and *P* values were adjusted for multiple hypothesis testing using the Bonferroni approach. We performed three versions of this analysis: (i) using all loops tested for variability (*n* = 85,294) and all histone peaks (*n *= 288,711) (*P*_All vs Contained _= 6.5 × 10^−212^, *P*_All vs Promoter _= 2.1 × 10^−260^, *P*_All vs Promoter-enhancer _= 1.9 × 10^−268^, *P*_Promoter vs Promoter-enhancer _= 1.0), (ii) using all loops tested for variability and histone peaks with variable activity. Variability in H3K27ac was assessed using the procedure outlined in ‘Identifying variable loops’. We set a threshold of FDR < 1% to define the set of variable histone peaks (*P*_All vs Contained _= 6.5 × 10^−212^, *P*_All vs Promoter _= 2.1 × 10^−260^, *P*_All vs Promoter-enhancer _= 0, *P*_Promoter vs Promoter-enhancer _= 4.9 × 10^−20^). (iii) using all variable loops (FDR < 10%) and all histone peak with variability activity (*P*_All vs Contained _= 2.2 × 10^−119^, *P*_All vs Promoter _= 1.9 × 10^−141^, *P*_All vs Promoter-enhancer _= 3.4 × 10^−13^, *P*_Promoter vs Promoter-enhancer _= 2.7 × 10^−26^). Taken together, these analysis indicate a stronger link between loop interaction frequency and gene expression when the loop is making direct contact with the gene’s promoter or when linking and enhancer to the promoter. Subsetting either loops or enhancers based on variability does not appear to improve the results.

Finally, we analysed if there was an enrichment for positive loop-gene correlation coefficients for the four maps described above. We tabulated the number of positive and negative coefficients for actual and randomized loop-gene pairs and assessed significance using Fisher’s exact test.

### Identifying group-specific loops

All analysis was performed on the set of loops tested for variability (*n* = 85,294). For each group (blood, embryonic, and solid-tissue-derived), we identified a set of loops that were present only in their member cell lines (Group_query_) and that did not differ between the other two groups (Group_other1_, Group_other2_) as follows: 1. Compute three sets of *t*-statistics based on the following three pairwise comparisons: interaction frequencies (normalized interaction frequency) for all cell lines in Group_query_ versus Group_other1_ (*t*_1_), interaction frequencies for all cell lines in Group_query_ versus Group_other2_ (*t*_2_), and interaction frequencies for all cell lines in Group_other1_ versus Group_other2_ (*t*_3_). 2. Rank order each vector of *t*-statistics in descending order. 3. Define three sets of loops (*T*_1_, *T*_2_, *T*_3_) such that their respective *t*-statistics are in the top 10% of *t*_1_, *t*_2_, and *t*_3_, respectively. 4. Define the final set of group-specific loops as $({T}_{1}\cap {T}_{2})-{T}_{3}$.

In this way, we specifically identified loops with a high interaction frequency in the group of interest compared to the other two groups and no difference between the other two groups.

### Annotating different DUEs

We used bioconductor´s package DEXSeq^77^ to identify DUEs. In brief, we flattened the Gencode (release 25; lifted to GRCh37 coordinates) file with parameters ‘-r no’ and used a modified script to extract counts with subRead (parameters -f -O -s 2 -p -T 40) as described in the vignette^78^. We classified the RNA-seq libraries either according to the three clusters identified with the PCA as described above, or by cell line (*n* = 22). Next we normalized for library size and dispersion, tested for DUEs, and estimated the exon log_2_-fold changes between (a) solid vs blood and stem cell-like vs blood, or (b) by cell type vs the median exon abundance. In this way, we identified (a) 95,137 and (b) 39,832 DUEs (FDR = 10%).

### Defining intragenic loops

As a way to identify intragenic loops that go from promoters to gene bodies, we followed the methods described previously^51^. Starting from the Gencode annotation (release 25; lifted to GRCh37 coordinates), we only kept protein-coding genes with at least one middle exon. We also removed all exons that overlapped previously defined CAGE peaks^79^. Based on visual inspection, we defined the promoter window as ±1 kb from the TSS and the upstream window as −5 kb from the 5′ exon boundary. We then identified intragenic loops as those loops for which one anchor fell in the promoter and the second in the upstream window of the same gene. In this way, we identified 1,372 loops within 1,074 genes. From this set, we identified exon–loop pairs (real pairs) by associating an exon with an anchor of an intragenic loop within 5 kb of their 5′ boundaries.

### Correlation of exon and loop anchors

We kept unique exon–loop pairs and correlated the normalized counts of exon and anchor strength across the 22 cell lines. As a control, we permuted all exons 100 times, creating new exon–loop pairs. We also accounted for gene expression by correlating all other exons within the same ‘looping’ gene and removed any exons within 20 kb of the centre of the anchor (all pairs). Then we performed a Pearson correlation for all complete observations and depicted only the DUEs across the 22 cell lines. For the scatterplot, we used the three-group classification specified above and we tested for correlation between real pairs and all pairs of the DUEs.

### TF enrichment analysis

We obtained the genomic coordinates for motif matches for 598 TFs from a previously published study^53^. For each TF, we tabulated the following four numbers: (i) the number of group-specific loop-ends overlapping a motif location, (ii) the number of group-specific loop-ends not overlapping a motif location, (iii) the number of non group-specific loop-ends overlapping a motif location, and (iv) the number of non group-specific loop-ends not overlapping a motif location. We assessed the significance of enrichment using a two-sided Fisher’s exact test. In cases in which any of values (1)–(4) were less than 5, we excluded this TF from further analysis. *P* values were corrected for multiple hypothesis testing using the Benjamini–Hochberg procedure. We repeated the analysis using different rank thresholds used to define the set of group-specific loops. Using a 5% threshold, we observed high correlation of fold-enrichment values (*r*_Blood _= 0.89, *r*_Embryonic _= 0.88). Moreover, out of the 120 significant TF enrichments for the blood-specific loops (FDR < 0.1), 74 were significant at this new threshold (3.74 fold-enrichment, *P* = 5.5 × 10^−41^ via hypergeometric test). For the 89 significant TF enrichment (FDR < 0.1) from embryonic-specific loops, 39 were significant at this new threshold (5.6 fold-enrichment, *P* = 1.1 × 10^−28^ via hypergeometric test). Using a 20% threshold, we again observed high correlation of fold-enrichment values (*r*_Blood _= 0.83, *r*_Embryonic _= 0.86). Moreover, out of the 120 significant TF enrichments (FDR < 0.1) for blood-specific loops, 95 were significant at this new threshold (2.72 fold-enrichment, *P* = 1.85 × 10^−38^ via hypergeometric test). For the 89 significant TF enrichments (FDR < 0.1) for embryonic-specific loops, 75 were significant at this new threshold (3.06 fold-enrichment, *P* = 1.5 × 10^−34^ via hypergeometric test).

### Transcription factor footprinting in ATAC–seq data

ATAC–seq data were processed (Methods) for signal tracks. Motifs for each TF were intersected with the loop annotations and ATAC–seq data were averaged across all motif instances using a custom Python script. Averaged signal was compared between blood-specific, embryonic-specific, and all loops, and the relevant ratios were computed and plotted for a given TF.

### GO biological process enrichment of group-specific loops

Using the procedure outlined in ‘Identifying group-specific loops’ above, we defined 3,384 blood-specific loops, 2,894 embryonic-specific loops, and 2,215 ‘misc’-specific loops. For each loop, we defined its ‘coordinates’ as the midpoint of loop end 1 to the midpoint of loop end 2. All three sets of loop-coordinates (blood, embryonic, and misc.) were examined for GO enrichment using the GREAT^66^ web tool with default options (version 3.0) (Supplementary Table 6).

### GWAS analysis

To test for enrichment of GWAS variants in our peak sets, we used all GWAS data sets in the GRASP database^61^ (*n* = 178). The GWAS SNPs were pruned to contain no variants in linkage disequilibrium by keeping the most significant *P* value where there were multiple linked variants for the same trait. We only kept GWAS with at least 1,000 SNPs after pruning in the analysis for sufficient quality to calculate an enrichment (*n* = 86). The set of pruned SNPs was then expanded to all linked variants with European *r*^2^ ≥ 0.8 for all further analysis.

We performed a rank-based enrichment of GWAS variants in each set of group-specific loops. We segmented each GWAS study into bins that represented decreasing tiers of significance. We set a minimum bin size of 50 and filled the first bin with the 50 most significantly associated variants for each study. We then filled the next bins with 2 × 50, 4 × 50 and 8 × 50 variants and then segmented the remaining variants into bins at the four quartiles of the remaining *P* value distribution. We used the pruned set of SNPs to set the bin thresholds. We then computed the rank fold change enrichment of peaks across the segmented GWAS^80^. For each bin we computed the fraction of GWAS variants that were less than or equal to the bin’s *P* value threshold that overlapped the loop regions. We calculated the fold change enrichment by dividing this fraction by the fraction of all GWAS variants of any significance level that overlapped our regions. Baseline enrichment is 1, which indicates no change from the base rate of overlap of all significant and non-significant variants in the study. An enrichment less than 1 means the most significant variants are depleted relative to the baseline and any value greater than 1 indicates that significant variants are enriched. To compute the significance of these enrichments, we permuted the *P* value associated with each GWAS SNP in the study 200 times and re-computed the enrichment relative to baseline. The empirical *P* value indicates the number of permuted studies for which the true study has a greater enrichment for the most significant bin of GWAS hits.

To compare the enrichment of each given GWAS study between sets of regions, we computed the total number of pruned genome-wide significant (*P* < 10 × 10^−8^) SNPs that overlapped each set of peaks and the total number that did not. An overlap was counted if any SNP in LD with the pruned SNP overlapped the regions of interest. This is important as we do not know which is the causal SNP. We then used Fisher’s exact test to statistically compare the rate of overlap between the two studies and to determine whether a set of regions was statistically enriched relative to another (Supplementary Table 7).

### LD score regression

Partitioned LD score regression (LDSC) is a method to determine whether there is an enrichment of GWAS effect sizes in a given portion of the genome^62^. We used LDSC to test whether our loop anchors, called loops, and DNase peaks within called loops that changed between cell types were associated with GWAS signal of complex traits. Using publicly available summary statistics of GWAS for complex traits^63^, we ran LDSC with the standard 1000G Phase III derived LD scores and weights, correcting for the baseline annotations (which contain the union of H3K27ac marked regions in the genome, H3K4me3 marked regions, and so on^62^ and the full set of Rad21-bound looped regions genome-wide. Regression coefficients were estimated using the overlap-annot option to partition effects across overlapping regions^62^ and with frequency files derived from 1000G Phase III Europeans and filtered for SNPs with minor allele counts of at least five. The following command was used: ldsc.py–h2< input summary statistics>–ref-ld-chr <1000G_EUR_Phase3_baseline>,<tested anchor regions>,<all rad21 peaks>–w-ld-chr < weights_hm3_no_hla>–overlap-annot–out < output estimates>–frqfile-chr <1000G.mac5eur>. Results were parsed for the enrichment of the tested anchor region and the reported statistics are taken directly from the command output.

### Correction for super-enhancers and cell type effects in LDSC

Super-enhancers are associated with increased chromatin looping and also with GWAS enrichment, so we wanted to test whether our signal was due to a super-enhancer signal. As such, we excluded called super-enhancers from any cell type from the tested anchor and loop annotations and re-ran the enrichment. In addition, after filtering out anchors from any loops that overlapped with super-enhancers we still see enrichment for the same traits (Extended Data Fig. 7, Supplementary Table 8). To assess whether the signal we observed might be just attributable to active chromatin in the cell types of interest, we added in all ten cell-type group annotations as covariates to the regression, along with the Roadmap control signal for per-mark accounting as previously described^67^ (Extended Data Fig. 7, Supplementary Table 8). The resulting regression was: ldsc.py–h2 {input.path}–ref-ld-chr <1000G_EUR_Phase3_baseline>,<tested anchor regions>,<all rad21 peaks>,<roadmap control>,<cell_type_group 1>,<cell_type_group 2>,<cell_type_group 3>,<cell_type_group 4>,<cell_type_group 5>,<cell_type_group 6>,<cell_type_group 7>,<cell_type_group 8>,<cell_type_group 9>,<cell_type_group 10>–w-ld-chr < weights_hm3_no_hla>–overlap-annot–out < output estimates>–frqfile-chr <1000G.mac5eur>.

### Reporting summary

Further information on research design is available in the Nature Research Reporting Summary linked to this paper.

## Online content

Any methods, additional references, Nature Research reporting summaries, source data, extended data, supplementary information, acknowledgements, peer review information; details of author contributions and competing interests; and statements of data and code availability are available at 10.1038/s41586-020-2151-x.

## Supplementary information

Reporting Summary

Supplementary Table 1 | Studied Cell types Cell types used in this study including the germ layer they are derived from and overlap with cell types in the Roadmap Epigenomics Mapping Consortium^47^ .

Supplementary Table 2 | Cell types and corresponding number of sequencing reads For each cell type and replicate the source, growing conditions and number of sequencing reads for ChIA-PET, ChIP-Seq and RNA-Seq are listed.

Supplementary Table 3 | RAD21 peaks (anchor regions) Peaks for RAD21-ChIA-PET data were called using MACS2. Peak calls across all samples were combined and overlapping peaks were merged.

Supplementary Table 4 | Pan-cell type cohesin-mediated chromatin loops List of 124,830 interactions identified across all 24 cell types studied, indicating which ones were tested for variability, which ones are variable, and which are present in a given cell type (# PETs > 4).

Supplementary Table 5 | H3K27ac peaks Peaks for H3K27ac-ChIP-Seq data for 22 out of 24 cell types were called using MACS and then merged to generate a list of 288,711 genome-wide peaks.

Supplementary Table 6 | GREAT GO Biological Process Enrichments All three sets of loop-coordinates (blood [n=3384 loops], embryonic [n=3894 loops], and misc. [n=2215 loops]) were examined for GO enrichment using the GREAT web tool with default options (version 3.0).

Supplementary Table 7 | Relative GWAS Enrichments blood vs. embryonic GWAS relative Enrichment p-values for blood (n=3384 loops) vs. embryonic (n=3894 loops) specific loops using a two-sided Fisher’s Exact Test. A total of 86 diseases were examined.

Supplementary Table 8 | GWAS LD-score regression analysis LD regression results for associations between GWAS traits and the tested groups of blood specific (n=3384 loops) an. embryonic (n=3894 loops) specific loops using a block jackknife t-test (n=1,100,000 HapMap3 SNPs) (s. Methods).

Supplementary Table 9 | Statistics for figure panels This Supplementary Table lists summary statistics, p-values and enrichment values for figure panels.

Supplementary Table 10 | Available data sets This Supplementary Table lists all data sets that have been generated for this study and where they can be obtained.

## Data Availability

The ChIA–PET data have been deposited on the ENCODE webportal and can be accessed here: https://www.encodeproject.org/publications/8d853642-45b4-47cf-ada6-f32c3058a39d/. The remaining data have been deposited in the GEO database under accession number GSE134745. There are no restrictions on data availability. Supplementary Table 10 lists all available data sets.

## Code availability

Scripts for processing the raw data can be found here: https://github.com/rohith-srivas/ChiaPET. Custom scripts for generating figures can be obtained upon request.
