# Supplementary material for: Landscape of cohesin-mediated chromatin loops in the human genome
Source: Nature. 2020 Jul 29;583(7818):737–43. doi: 10.1038/s41586-020-2151-x (PMC7410831; doi:10.1038/s41586-020-2151-x)
Supplement: Supplementary file 1 — Reporting Summary [file 41586_2020_2151_MOESM1_ESM.pdf]

nature research

Corresponding author(s): Michael P. Snyder

Last updated by author(s): Sep 9, 2019

Reporting Summary

Nature Research wishes to improve the reproducibility of the work that we publish. This form provides structure for consistency and transparency in reporting. For further information on Nature Research policies, see Authors & Referees and the Editorial Policy Checklist.

Statistics

For all statistical analyses, confirm that the following items are present in the figure legend, table legend, main text, or Methods section.

n/a	Confirmed	
☐	☒	The exact sample size (*n*) for each experimental group/condition, given as a discrete number and unit of measurement
☐	☒	A statement on whether measurements were taken from distinct samples or whether the same sample was measured repeatedly
☐	☒	The statistical test(s) used AND whether they are one- or two-sided<br>*Only common tests should be described solely by name; describe more complex techniques in the Methods section.*
☐	☒	A description of all covariates tested
☐	☒	A description of any assumptions or corrections, such as tests of normality and adjustment for multiple comparisons
☐	☒	A full description of the statistical parameters including central tendency (e.g. means) or other basic estimates (e.g. regression coefficient) AND variation (e.g. standard deviation) or associated estimates of uncertainty (e.g. confidence intervals)
☐	☒	For null hypothesis testing, the test statistic (e.g. *F*, *t*, *r*) with confidence intervals, effect sizes, degrees of freedom and *P* value noted<br>*Give P values as exact values whenever suitable.*
☒	☐	For Bayesian analysis, information on the choice of priors and Markov chain Monte Carlo settings
☐	☒	For hierarchical and complex designs, identification of the appropriate level for tests and full reporting of outcomes
☐	☒	Estimates of effect sizes (e.g. Cohen's *d*, Pearson's *r*), indicating how they were calculated

Our web collection on statistics for biologists contains articles on many of the points above.

Software and code

Policy information about availability of computer code

Data collection	All analysis was done in R (version 3.3.1) using custom scripts which can be found here: https://github.com/rohith-srivas/ChiaPET
Data analysis	All analysis was done in R (version 3.3.1) using custom scripts which can be found here: https://github.com/rohith-srivas/ChiaPET

For manuscripts utilizing custom algorithms or software that are central to the research but not yet described in published literature, software must be made available to editors/reviewers. We strongly encourage code deposition in a community repository (e.g. GitHub). See the Nature Research guidelines for submitting code & software for further information.

Data

Policy information about availability of data

All manuscripts must include a data availability statement. This statement should provide the following information, where applicable:

- Accession codes, unique identifiers, or web links for publicly available datasets
- A list of figures that have associated raw data
- A description of any restrictions on data availability

The ChIA-PET data has been deposited on the ENCODE webportal and can be accessed here: https://www.encodeproject.org/publications/8d853642-45b4-47cf-ada6-f32c3058a39d/
The remaining data have been deposited in the GEO database under accession number GSE134745. There are no restrictions on data availability.

Field-specific reporting

Please select the one below that is the best fit for your research. If you are not sure, read the appropriate sections before making your selection.

☒ Life sciences ☐ Behavioural & social sciences ☐ Ecological, evolutionary & environmental sciences

For a reference copy of the document with all sections, see nature.com/documents/nr-reporting-summary-flat.pdf

Life sciences study design

All studies must disclose on these points even when the disclosure is negative.

Sample size	No sample-size calculation was performed.
Data exclusions	For two cell lines we were unable to produce RNA-seq and H3K27ac ChIP-seq data due to lack of material. This exclusion was not pre-established. For integrative analysis we have utilized only 22/24 cell lines.
Replication	We have performed all experiments in biological replicates to ensure reproducibility of the data. The data passes ENCODE QC metrics; these analysis have been detailed in our Supplementary figures.
Randomization	There are no experimental groups in this study. We have assigned random identifiers to samples during the experimental procedures to minimize batch effects.
Blinding	investigators were not blinded, but samples were stripped of their ID and assigned a random ID at the beginning of the respective experiments.

Reporting for specific materials, systems and methods

We require information from authors about some types of materials, experimental systems and methods used in many studies. Here, indicate whether each material, system or method listed is relevant to your study. If you are not sure if a list item applies to your research, read the appropriate section before selecting a response.

Materials & experimental systems

n/a	Involved in the study	
☐	☒	Antibodies
☐	☒	Eukaryotic cell lines
☒	☐	Palaeontology
☒	☐	Animals and other organisms
☒	☐	Human research participants
☒	☐	Clinical data

Methods

n/a	Involved in the study	
☐	☒	ChIP-seq
☒	☐	Flow cytometry
☒	☐	MRI-based neuroimaging

Antibodies

Antibodies used	Abcam Anti-RAD21 antibody (ab992, lot ID:GR184716) and H3K27ac (Abcam #4729, lot ID: GR104852)
Validation	Abcam Anti-RAD21 antibody (ab992, lot ID:GR184716) https://www.encodeproject.org/antibodies/ENCAB529YRC/ and H3K27ac (Abcam #4729, lot ID: GR104852) https://www.encodeproject.org/antibodies/ENCAB000BSK/)

Eukaryotic cell lines

Policy information about cell lines

Cell line source(s)	K1 Sigma-Aldrich<br>HepG2 ENCODE<br>ARPE-19 ATCC<br>JURKAT ATCC<br>MCF7 ENCODE<br>DU145 ATCC<br>NCI-H1437 ATCC<br>HT-1376 ATCC<br>MSLCL established in the Snyder lab, Stanford University<br>SU-DHL-2 ATCC<br>SU-DHL-4 ATCC<br>GM12878 ENCODE

	LNCaP ATCC<br>HPAEC ATCC<br>MSFIB established in the Snyder lab, Stanford University<br>H1-hESC (WA01; WiCell)<br>hTERT-HME1 ATCC<br>MSiPS established in the Snyder lab, Stanford University<br>LX differentiated from H9-hESC in the Dalton lab, UGA<br>NP differentiated from H9-hESC in the Dalton lab, UGA<br>H9-hESC Dalton lab, UGA<br>KU-19 DSMZ<br>K562 ENCODE<br>HT-1197 ATCC
Authentication	Cell lines were not authenticated. Sequencing libraries from the same cell line were checked for proper genotype as determined by WGS
Mycoplasma contamination	Cell lines were not tested for mycoplasma
Commonly misidentified lines<br>(See ICLAC register)	K1 (Thyroid, papillary carcinoma) is commonly misidentified with CVCL_9918 another Thyroid, papillary carcinoma. Since both cell are papillary carcinoma the conclusions of our study would not be affected

ChIP-seq

Data deposition

☒ Confirm that both raw and final processed data have been deposited in a public database such as GEO.

☒ Confirm that you have deposited or provided access to graph files (e.g. BED files) for the called peaks.

Data access links<br>*May remain private before publication.*	The ChIP-Seq data from this study have been deposited in the GEO database under accession number GSE134745.
Files in database submission	A full list of file can be found under the accession numbers provided above.
Genome browser session<br>(e.g. UCSC)	no longer applicable

Methodology

Replicates	two biological replicates per cell type
Sequencing depth	we obtained on average 43 ±9 million paired-end reads 2*101bp) per sample.
Antibodies	Abcam Anti-RAD21 antibody (ab992, lot ID:GR184716) and H3K27ac (Abcam #4729, lot ID: GR104852)
Peak calling parameters	peaks were called using MACS2 (non-default parameters: -q 0.01).
Data quality	Two replicates were performed per cell line. ChIP data has been validated according to ENCODE standards, e.g. Relative strand correlation (RSC). Quality control metrics and concordance between replicates has been detailed in Extended data figure 1 and 2.
Software	All analysis was done in R (version 3.3.1) using custom scripts which can be found here: https://github.com/rohith-srivas/ChiaPET. Picard MarkDuplicates was used to remove duplicates. Peaks were called using MACS2.
